# The Tandem Reconnection and Cusp Electrodynamics Reconnaissance Satellites (TRACERS) Magnetic Control Plan

**DOI:** 10.1007/s11214-026-01326-2

**Published:** 2026-09-07

**Authors:** D. M. Miles, A. Lasko, M. Blandin, S. Bounds, R. Caron, A. Carton, J. Dolan, R. Dvorsky, M. G. Finley, A. Flores, C. Goss, K. Greene, J. Halekas, G. B. Hospodarsky, C. A. Kletzing, D. Mark, I. Miller, P. Nguyen, S. Omar, E. Orrill, R. Prasad, A. Rospos, A. Slagle, R. J. Strangeway, R. Torbert, R. Vernot, A. Washington

**Affiliations:** 1 Department of Physics and Astronomy, The University of Iowa, Iowa City, IA USA; 2 Department of Earth, Planetary and Space Sciences, University of California Los Angeles, Los Angeles, CA USA; 3 Geospace Physics Laboratory, NASA Goddard Space Flight Center, Greenbelt, MD USA; 4 Department of Astronomy, University of Maryland, College Park, MD USA; 5 Millenium Space Systems, A Boeing Company, El Segundo, CA USA; 6 Bison Aerospace, Wyoming, USA; 7 Nextage, LLC, Glenwood, Maryland, USA; 8 Space Sciences Laboratory, University of California, Berkeley, CA USA; 9 University of New Hampshire, Durham, NH USA

**Keywords:** Magnetometry, Magnetic cleanliness, TRACERS, MAGIC

## Abstract

The Tandem Reconnection And Cusp Electrodynamics Reconnaissance Satellites (TRACERS) mission implemented a magnetics control plan to help meet its science objectives. TRACERS studies the variability of magnetic reconnection using two closely spaced spacecraft taking high-cadence plasma measurements through the northern magnetospheric cusp. Science measurements of the local AC and DC magnetic field and medium energy electrons can be degraded by local stray magnetic fields from the spacecraft and instruments. The measured low frequency and static magnetic field can be directly contaminated by static magnetic fields from magnetised or ferromagnetic materials while the AC magnetic fields are susceptible to contamination from time varying electrical currents. Incoming electrons can be deflected by local magnetic fields altering their apparent incoming direction at detection. TRACERS uses a combination of source control, physical separation, magnetic screening, and post-hoc signal processing to ensure it meets its measurement objectives.

## Background and Motivation

The Tandem Reconnection And Cusp Electrodynamics Reconnaissance Satellites (TRACERS) mission (Miles et al. 2025b; Petrinec et al. 2025) studies the variability of magnetic reconnection using two closely spaced spacecraft taking high-cadence plasma measurements through the northern magnetospheric cusp. The TRACERS science objectives require high-fidelity measurements of the local DC magnetic field by the MAG fluxgate magnetometer instrument (Strangeway et al. 2025) and the MAGIC technology demonstration (Miles et al. 2025a), the AC magnetic field by the MSC magnetic search coil instrument (Hospodarsky et al. 2025) and medium energy electrons by the ACE instrument (Halekas et al. 2025). These measurements can be degraded by local stray magnetic fields from the spacecraft or its subsystems and instruments. The measured magnetic field can be directly contaminated by static magnetic fields from magnetised or ferromagnetic materials while the AC magnetic fields can be contaminated by time varying electrical currents. Incoming electrons can be deflected by local magnetic fields altering their apparent incoming direction at detection. TRACERS implemented a magnetics control plan to limit the local stray magnetic field through source control and implemented screening activities to understand the as-built remaining magnetic sources and enable ground-based data post-processing mitigation.

TRACERS defined overlapping frequency ranges for AC and DC magnetic fields based upon the science performed by the two magnetic field instruments – zero to 50 Hz for the fluxgate magnetometer (Strangeway et al. 2025) and 1 to 1000 Hz for the search coil (Hospodarsky et al. 2025). The MAGIC technology demonstration fluxgate (Miles et al. 2025a) is also susceptible to interference from zero to 50 Hz, but it does not impose any requirements on the TRACERS mission because it is a do-no-harm technology demonstration.

The magnetic field generated by an object decays rapidly with distance so the first mitigation strategy for magnetic cleanliness is simply separating the susceptible sensors from the most significant magnetic sources. The fluxgate and search coil sensors were each intended to be deployed 100 cm from the spacecraft on two-segment non-magnetic booms. However, a failure of the initial deployable boom during environmental testing necessitated a design change to a 70 cm fixed bracket late in the mission. Consequently, the TRACERS magnetics control plan is designed around evaluating and controlling sources based on their stray field at a distance of 100 cm but the impact of these sources increased when the magnetometer sensors were moved closer to the spacecraft (Sect. 7).

The electron instrument is susceptible to magnetic fields at its aperture at frequencies below a local electron ∼1 MHz gyrofrequency. Stray magnetic fields near the ACE sensor will lead to deflection of lower-energy electrons, which adversely affect the angular resolution of the instrument.

## Design Guidelines

It is generally not practical to completely avoid the use of components that generate hard permanent fields; however, TRACERS used a preferred/discouraged materials list (the Appendix) to guide part selections. The fields resulting from such materials are usually quite stable and can sometimes be partially cancelled by serendipitous or intentional positioning relative to other magnetic components. This creates a higher-order source, as experienced by a distant sensor, whose stray field drops off more rapidly. For example, latching relays are inherently magnetic due to the internal permanent magnet required for operation. However, they can be arranged in mirrored pairs so the combined stray field is partially cancelled. A small permanent compensating magnet properly sized and oriented on the device, can be used to cancel out the field of the item’s internal magnet. However, compensation magnets were not employed on TRACERS due to the potential to create strong local magnetic fields that could influence the ACE electron instrument that is mounted on a spacecraft face sheet or require additional risk reduction testing to spacecraft components to ensure they were not impacted by adding local magnetic fields.

The battery was located near the center of the spacecraft body, as far as possible from the magnetometer sensors. The battery design has an asymmetric string-cell configuration that generates a stray magnetic field dependent on the battery charge/discharge current. Two batteries were therefore arranged back-to-back to reduce the magnetic moment in two axes. The latch valve assemblies were oriented in opposite directions to maximize magnetic dipole cancellation in two components. Heaters used throughout the spacecraft were designed and fabricated to ensure that current trace paths were generally self cancelling.

TRACERS electrical design guidelines were intended to minimize stray fields due to uncompensated current loops or stray current paths. TRACERS used balanced twisted pairs to provide dedicated return currents in all harnessing to minimise open current loops. Special attention was placed on subsystems that, based on theory or prior mission experience, had the potential for large and/or time varying magnetic signatures. The solar panels were back-wired (e.g., Stern and DeLapp 2004; Stern et al. 2013) such that the interconnects were anti-parallel and the bulk current flow in each panel was generally self-cancelling. The TRACERS integration schedule did not provide an opportunity to characterize the effectiveness of the back-wiring prior to launch.

Electronics subsystems were required to implement a local star grounding design to minimize stray current from ground return loops. Similarly, instruments and subsystems were required to return current via balanced twisted pairs and were forbidden from using chassis ground for current return.

Special rules applied to the zone around all magnetometer sensors – in practice defined by the magnetometer brackets and ending at the bulkhead mount to the spacecraft. The brackets were designed from non-ferromagnetic materials including aluminum, titanium, carbon fiber composites, and PEEK engineering plastic. Specialty connectors constructed from titanium and aluminium were used when field joints were required on the brackets. Special attention was given to the grounding strategy of the multilayer insulation (MLI) blanketing the magnetometer brackets to avoid creating open loops in the ground wire.

## Component and Assembly Level DC Magnetic Field Screening

Four different techniques were used by the different participating institutions to complete DC magnetic field screening of piece parts and assemblies.

**Fig. 1:**
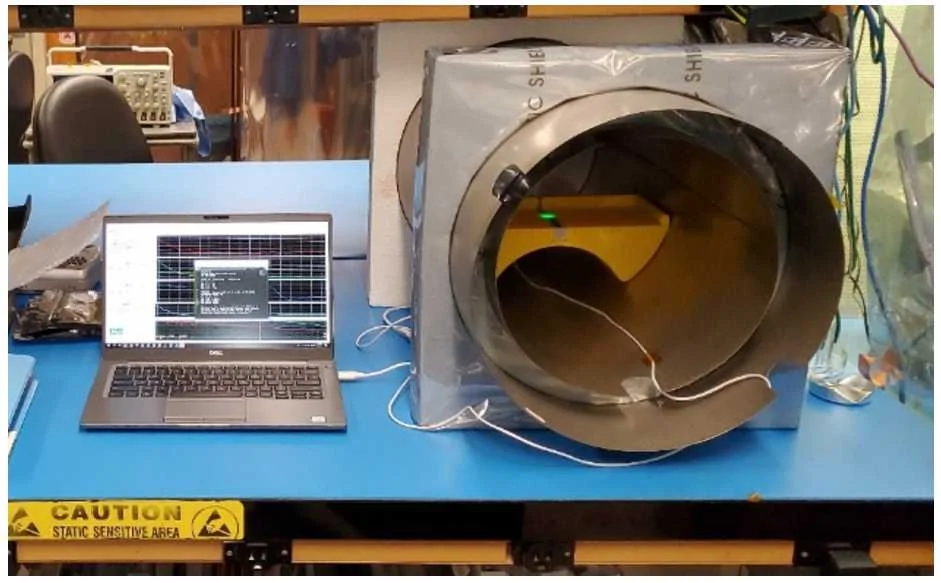
Mumetal shield used to screen components and piece parts by measuring their stray field at varying distances

The MAG (Strangeway et al. 2025) fluxgate magnetometer instrument team used a magnetometer centered in a mumetal shield (Fig. 1) to directly measure the stray field of test articles that were moved backwards at fixed intervals. This test was carried out three times – one for each component axis.

**Fig. 2:**
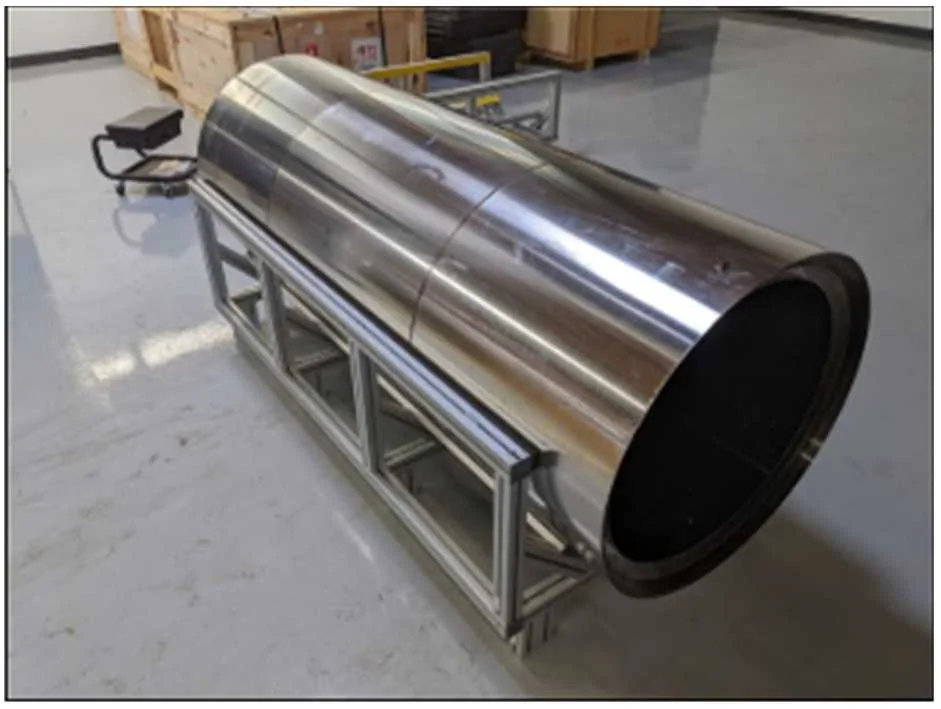
Equivalent but much larger three-layer mumetal shield assembly used to screen spacecraft subsystems and components in the same manner

The spacecraft provider used a similar, but much larger, three-layer mumetal shield assembly to screen spacecraft components and subassemblies (Fig. 2) in the same manner.

**Fig. 3:**
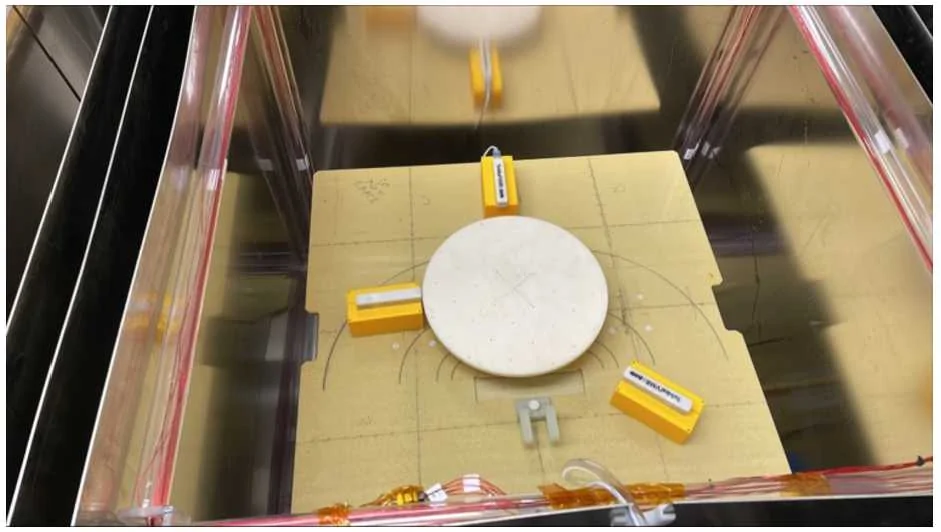
Semiautomated magnetic screening apparatus where test articles are rotated at a fixed frequency, the stray field is measured at three distances using quantitative spectral analysis, and a dipole is fit to the measurements (Dorman et al. 2024)

The mission team developed a semi-automated tool (Fig. 3) for screening components and piece parts which is documented in Dorman et al. (2024). A one meter cubic three layer shield assembly leftover from a previous mission, was modified with a magnetically clean rotating plate and three magnetometers at varying distances. Test articles were rotated at a fixed frequency, the modulated field measured using quantitative spectral analysis, and a curve fit to the three measurements at three distances to estimate the dipole moment.

Final instrument assemblies were screened as they were delivered for instrument suite integration by manually waving them a fixed distance from a stationary magnetometer. This provided a quick check of the stray field of complete assemblies which could be completed with minimal infrastructure in a clean room.

## DC Stray Field Allocation

The goal of the DC magnetic control plan was not to predict the stray field that would be experienced by sensitive instruments such as the magnetometers but rather to ensure that the final stray field was less than the mission allowable maximum value of 100 nT at the MAG sensor. The goal was to efficiently approve flight objects that posed no risk to the mission and identify potentially threatening items for consideration by the magnetics control board and either mitigation or the release of reserved stray field allocation.

Several assumptions were necessary to estimate the stray field that would result from the addition of many magnetic sources, most of which are not well known in advance. Inspired by the approach in Musmann (2010), a maximum stray magnetic field was allowed at two controlled locations – the fluxgate sensor and the aperture of the ACE electron instrument. Each magnetically screened object was compared to an allocation of the maximum allowable stray magnetic field by pro-rating against the object’s mass. That is, for a spacecraft of mass $m_{sc}$ and a maximum allowable stray magnetic field of $B_{\mathrm{limit}}$, then each object i is allocated a maximum allowed stray magnetic field contribution of $B_{i}$ based on its mass $m_{i}$ via

1 $$ \frac{B_{i}}{B_{\mathrm{limit}}} = \frac{m_{i}}{m_{sc}} $$

or

2 $$ \mathrm{B}_{i} = B_{\mathrm{limit}} \frac{m_{i}}{m_{sc}} $$

Allocating stray magnetic field by mass proportion is designed to be conservative and tend to over-estimate the aggregate field of many point sources. Since the relative orientations of the various magnetic sources are unknown, and generally unknowable until the spacecraft layout is complete, a random orientation was assumed such that, on average, the N possible magnetic sources in the spacecraft are assumed to add in quadrature such that the maximum expected stray field $\mathrm{B}_{\max}$ was:

3 $$ \mathrm{B}_{\max} = \sqrt{\sum _{i}^{N} \left ( B_{i} \right )^{2}} $$

Consequently, the magnetic field allocation should underestimate the final real value. For example, if the spacecraft is divided into two subsystems, 1 and 2, each with half the total spacecraft mass then the total stray field would be

4 $$ \mathrm{B}_{\max} = \sqrt{\left ( B_{1} \right )^{2} + \left ( B_{2} \right )^{2}} = \sqrt{\left ( B_{\mathrm{limit}} \frac{m_{\frac{sc}{2}}}{m_{sc}} \right )^{2} + \left ( B_{\mathrm{limit}} \frac{m_{\frac{sc}{2}}}{m_{sc}} \right )^{2}} $$

Simple manipulation then shows that

5 $$ \mathrm{B}_{\max} = \sqrt{\left ( \frac{B_{\mathrm{limit}}}{2} \right )^{2} + \left ( \frac{B_{\mathrm{limit}}}{2} \right )^{2}} = \frac{B_{\mathrm{limit}}}{\sqrt{2}} \approx 0.7 B_{\mathrm{limit}} $$

This effect is more pronounced as the spacecraft is divided into smaller pieces. Dividing into ten equal pieces gives worst case field of $\sim 0.32\ B_{\mathrm{limit}}$ and dividing into fifty equal pieces gives $\sim 0.14\ B_{\mathrm{limit}}$. Since it is unknown, a-priori, whether the allocation will be dominated by a few subsystems or whether it will be spread out more evenly, the budget does not assume the benefit of adding many small objects. The simple mass-proportional weighting accounts for the worst-case where the stray field is dominated by a small number of sources..

For planning purposes, it was assumed that some subsystems would violate their nominal allocation and may not be able to be mitigated via cancellation. For example, ferromagnetic objects such as springs cannot be easily compensated due to their changing geometry and commercially procured spacecraft subsystems were flown as-is. The magnetic control plan therefor needed to establish a reserve of un-allocated stray field that could be released on a case-by-case basis to account for systems that violated their nominal allocation and could not be easily mitigated. A reserve percentage $k_{\mathrm{reserve}} =0.5$ was held back from the pro-rating of the magnetic allocation such that at least half of the maximum allowable stray magnetic field of $B_{\max}$ was explicitly reserved for discretionary use by the magnetic control board.

For a spacecraft of mass $m_{sc}$ and a maximum allowable stray magnetic field of $B_{\max}$, each object i is screened against an acceptable stray magnetic field contribution of $B_{i,\mathrm{threshold}}$ based on its mass $m_{i}$ and the reserve percentage $k_{\mathrm{reserve}}$

6 $$ B_{i,\mathrm{threshold}} = k_{\mathrm{reserve}} B_{\mathrm{limit}} \frac{m_{i}}{m_{sc}} $$

Any object whose screened magnetic contribution was below that threshold was immediately deemed acceptable for flight. Objects whose screened magnetic contribution was above this threshold were brought to the magnetic control board for consideration of either mitigation by cancellation or the release of stray field reserve.

As an example, the TRACERS Instrument Suite (TIS) Main Electronics Box (MEB) had a measured mass of 6.6 kg and a measured stray field extrapolated to 1 m of 2.1 nT. The acceptable threshold for the MEG using Eq. (6) is $0.5\times 87\ \mathrm{nT}\ \times \frac{6.6\ \mathrm{kg}}{127\ \mathrm{kg}} =2.3\ \mathrm{nT}$. Since the measured stray field (2.1 nT) is below the control threshold (2.3 nT) the instrument was accepted for flight.

One disadvantage of the mass-weighting approach is that small items, such as piece parts, can fail their proportional mass-weighted allocation but have such a small absolute magnetic field that their contribution to the total is negligible and/or difficult to measure precisely. A de-minimis threshold was used to account for such parts so that, regardless of mass, if the measured stray field of an object extrapolated to less than 1 nT at one meter the part was acceptable. Additionally, a part that failed when screened individually was allowed to pass when screened as part of a higher-level assembly. This provided an efficient method to take advantage of opportunistic cancellation as assemblies were built up.

Objects that failed magnetic screening when extrapolated to 1 m were re-analyzed with their stray field extrapolated to their actual placed geometry in the spacecraft CAD model. This typically provided significant relief compared the nominal 1 m screening threshold which essentially assumes that all magnetic sources are placed at the skin of the spacecraft nearest to the magnetometer sensor. The evaluation using actual placed geometry was complete primarily before the change from a 1 m deployable boom to a 70 cm fixed bracket. The increase in apparent stray field due to this change was mitigated using post-processing as described in Sect. 8. Objects within 30 cm of ACE were also analyzed using ACE geometry and constraints to check for strong near-field effects on the incoming electrons.

The latch valves were found to have the largest stray magnetic field by a significant margin (Table 1). After consideration, they were dispositioned as ‘use-as-is’ rather than attempting magnetic cancellation to avoid creating a stronger and more complex field at the nearer ACE electron instrument. Their DC stray field at the fluxgate magnetometers will be mitigated via post-processed in-situ calibration.

**Table 1:** Magnetic screening results of selected instruments and spacecraft subsystems

Component	Worst Case Static (nT @ 1 m)	Powered (nT @ 1 m)	Notes	Disposition
ABBA	50	53	<15 nT using placed geometry	Use As Is
Latch Valves	500 (dual) 749 (single)		<93 nT using placed geometry	Use As Is
RIB	10 (reset) 22 (set)	10 (reset) 22 (set)	<7 nT using placed geometry	Use As Is Telemeter status
90° Thruster	14		<2 nT using placed geometry	Use As Is
Axial Thruster	11		<3 nT using placed geometry	Use As Is
MAG	0.2		Within self-allocation	Within Allocation
MSC	1.2		<0.044 nT using placed geometry	Within Allocation
ACI	0.4		De Minimis	Within Allocation
ACE	0.1		De Minimis	Within Allocation
MAGIC	0.2		< 1.6 nT using placed geometry Within on-boom allocation	Within Allocation
MSSS	0.068		De Minimis	Within Allocation
NAVI	0.005		De Minimis	Within Allocation
PAM	0.872		De Minimis	Within Allocation
PCU	0.069		De Minimis	Within Allocation
PIC2.0	0.307		De Minimis	Within Allocation
TAS1	0.142		De Minimis	Within Allocation
TAS2	0.141		De Minimis	Within Allocation
Enable Plugs	0.747		De Minimis	Within Allocation
Separation Springs	0.266		De Minimis	Within Allocation
Solar Cells	0.732		De Minimis	Within Allocation

Table 1 shows the screening results and disposition of selected instruments and spacecraft subsystems.

The ACE electron instrument is mounted on a spacecraft face-sheet, closer to the spacecraft subsystem stray field sources than the fluxgates, but can tolerate a considerably larger stray field of up to 800 nT. For simplicity, TRACERS defined a sensitivity region of 30 cm around the ACE aperture and checked the known non-trivial sources within this region.

## AC Screening

**Fig. 4:**
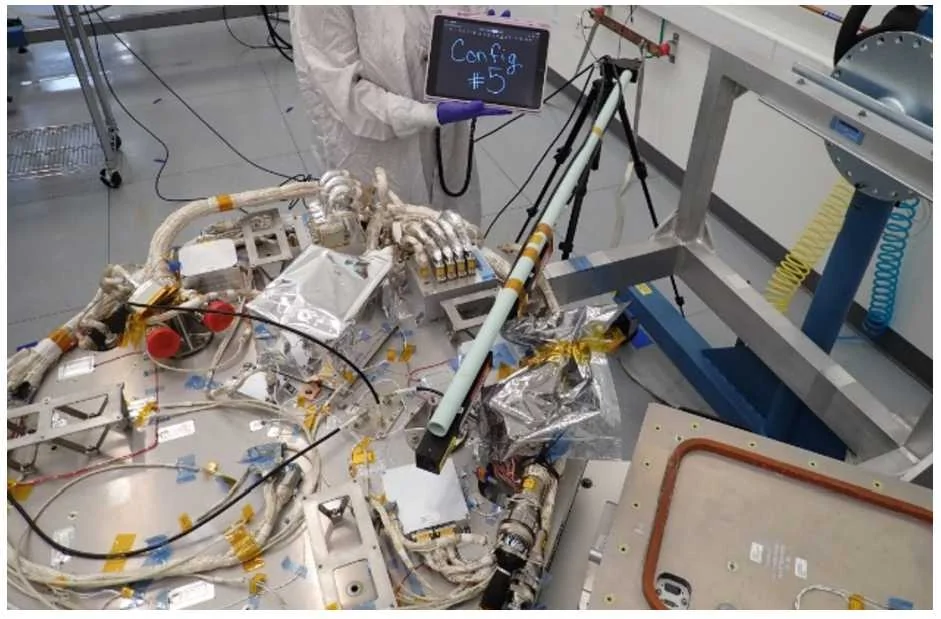
Example magnetic screening configuration - two magnetometers are mounted on a non-magnetic tube suspended over the spacecraft from a tripod. One sensor is immediately above the subsystem being screened and the other is 30 cm back

Several test campaigns of AC magnetic measurements were completed to identify sources that might need to be addressed in post-processed signal processing. Test campaigns prior to spacecraft closeout allowed magnetometers to be placed both near, and at some separation from, instrument and major spacecraft subsystem to create a database of potential interference and link it to its source. Two Bartington magnetometers were mounted 30 cm apart on a non-magnetic, carbon fiber pole that was mounted on a tripod so they could be located above each instrument of interest (Fig. 4). Each instrument or operational state was toggled on and off three separate times to ensure characterization of each instrumental impact.

**Fig. 5:**
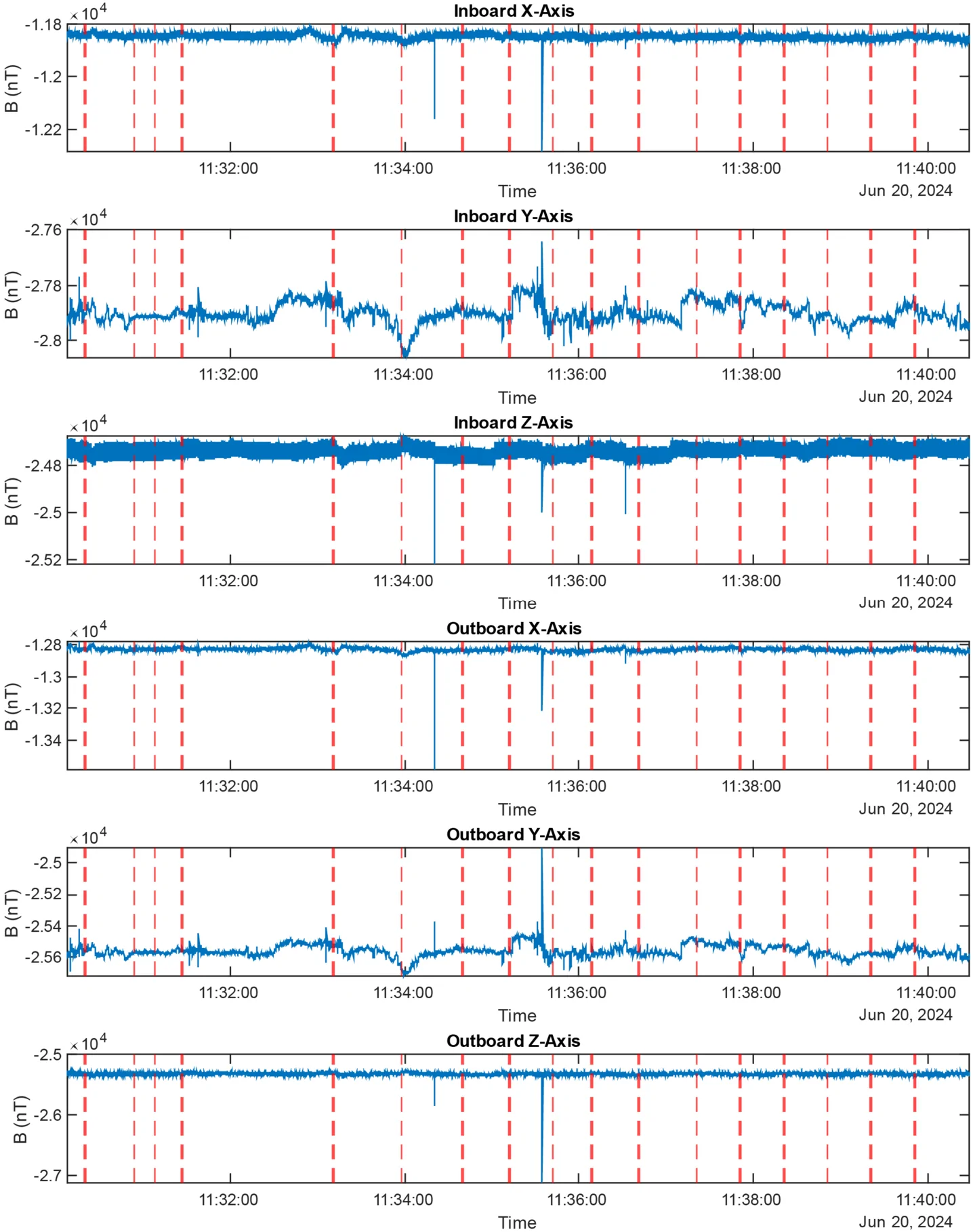
Raw vector magnetic field data taken while exercising the GPS subsystem of a TRACERS spacecraft. The stray field created by the subsystem is masked by significant local magnetic noise. Red vertical lines correspond to times from the spacecraft command log

The cleanroom housing the spacecraft integration was not magnetically clean so the fields of the objects being exercise was partially masked by large environmental variations. Figure 5 shows raw data captured while exercising the GPS subsystem; however, any resulting stray field variation is masked by environmental noise.

**Fig. 6:**
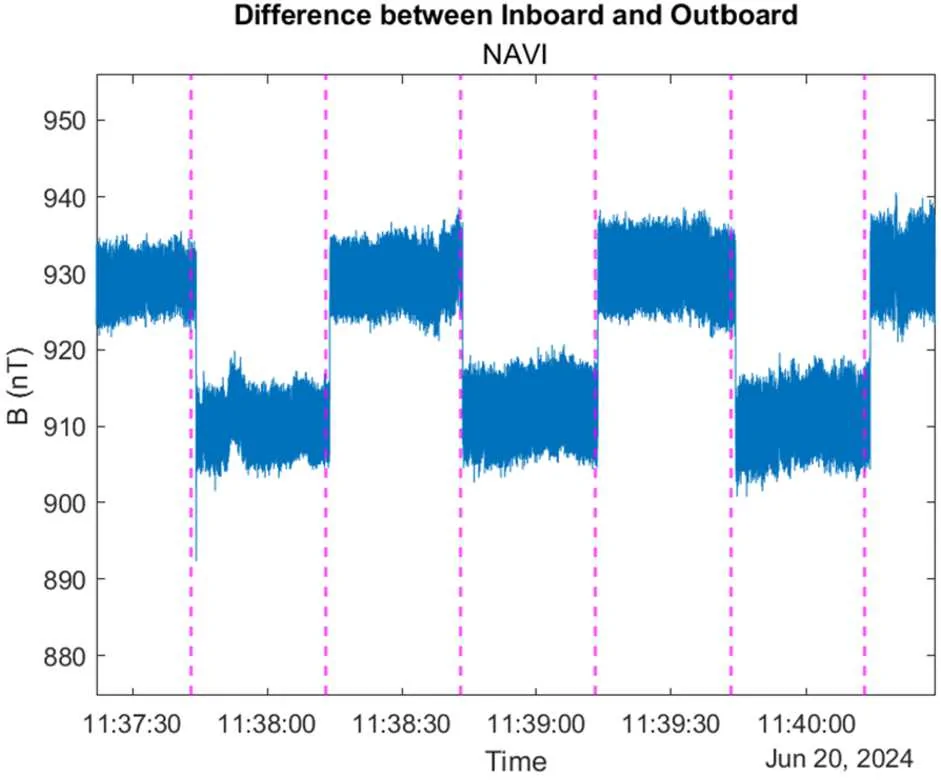
The stray field of the GPS (NAVI subsystem) was recovered using the difference in the scalar field between the two fluxgates, which shows clear ∼20 nT steps as the subsystem was toggled

The environmental field should be similar at both sensors, but the test object stray fields should decrease with distance as roughly a dipole $\left ( {1} / {d^{3}} \right )$ where the first sensor is placed at the source and the second sensor is 30 cm away. The gradient between the sensors therefore provides an effective tool to isolate the stray magnetic field as shown in Fig. 6.

The geometry of the measurement allows it to be roughly extrapolated to the locations of the sensitive science instruments (MAG, MAGIC, MSC). For MAG and MSC the article under test was assumed to be located at the center of the spacecraft, so the field was projected to an assumed spacecraft radius of 50 cm and an assumed mounting distance of 65 cm (accounting for the physical size of the sensor and assuming that the sensing element is the center of the sensor) for a total 115 cm separation using

7 $$ B_{\mathrm{MAG}/\mathrm{MSC}} = B_{30\text{ cm}} * \left ( \frac{30}{115} \right )^{3} $$

For MAGIC the article under test was assumed to be located at the center of the spacecraft, so the field was projected to an assumed spacecraft radius of 50 cm and an assumed mounting distance of 15 cm for a total 65 cm separation using

8 $$ B_{\mathrm{MAGIC}} = B_{30\text{ cm}} * \left ( \frac{30}{65} \right )^{3} $$

It is likely that higher order magnetic moments are also captured in the $B_{30cm}$ meaning that the impacts at the locations will be even smaller than estimated. Table 2 summarizes the screening data from the major spacecraft subsystems. The Relay Interface Board (RIB) has the largest state-dependent stray; however, its state does not change once on-orbit. The stray field from the battery discharge current will likely vary during the region of interest due to solar illumination and spacecraft state and will need to be mitigated using the techniques described in Sect. 8.2.

**Table 2:** Summary AC stray field from major spacecraft subsystems/modes extrapolated to the estimated location of MAGIC, MAG, and MSC

Instrument	ΔB (nT)	At MAGIC location (nT)	MAG/MSC location (nT)
PIC	75	7	1.5
Heater 204	1.5	0.15	0.02
Heater 103	1	0.1	0.007
NAVI (IMU)	∼ 5	∼0.5	0.08
NAVI (GPS)	20	2.0	0.3
PAM (non-hazard)	7	0.7	0.13
PAM (hazard)	3	0.2	0.035
RIB	9285	910	170
Battery Discharge	150	15	2.5

## Mitigating Fluxgate-Fluxgate Sensor Interactions

**Fig. 7:**
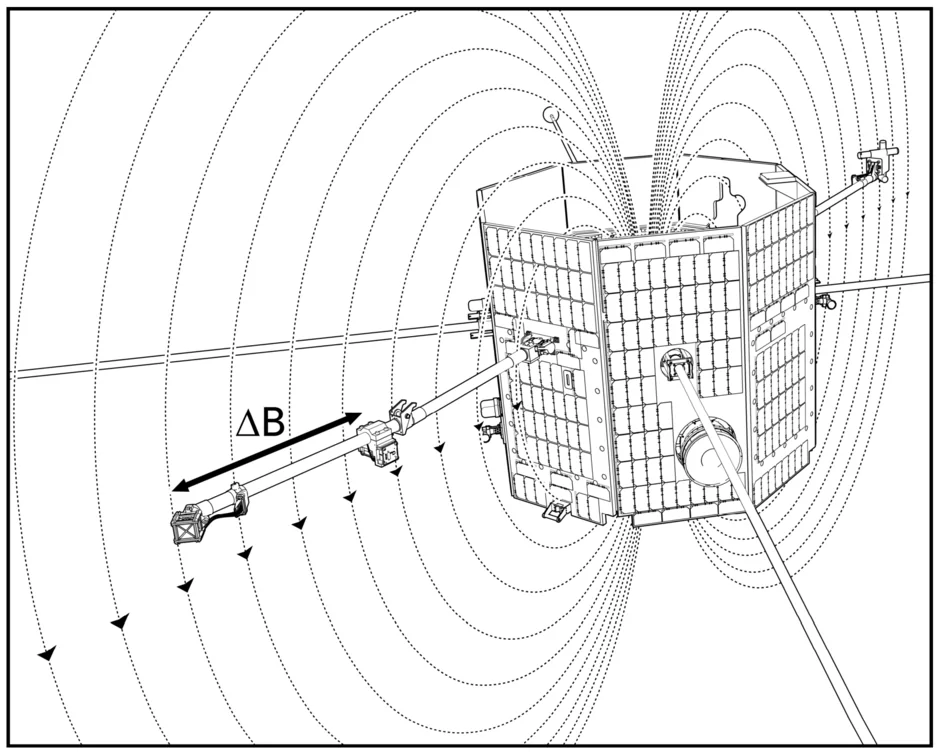
The two fluxgate instruments on the TRACERS spacecraft form an ad-hoc gradiometer enabling the common geophysical magnetic field to be separated from the stray field of the spacecraft that is observed at different strengths (ΔB) at the two instruments. However, their proximity also causes mutual interference

Magnetic gradiometers, comprising two magnetometers mounted at different distances from a spacecraft on a common boom (Fig. 7), are a common technique (e.g., Behannon et al. 1977; Lepping et al. 1995; Howell and Pappalardo 2020; Cheng et al. 2018; Russell et al. 1980) to help mitigate local spacecraft noise and provide instrument level redundancy. A variety of signal processing techniques ranging from model driven (e.g., Lepping and Ness 1978; Ness et al. 1971; Neubauer 1975) to decomposition (e.g., Finley et al. 2023a; Imajo et al. 2021) and feature tracking (e.g., Sen Gupta and Miles 2023) can be used to separate the desired geophysical field, which is common between the sensors, from the stray magnetic field generated by the spacecraft, subsystems, and instruments, whose amplitude diminishes with distance and is therefore observed at different amplitudes at the two sensors (Fig. 7, ΔB). However, implementing a dual-sensor gradiometer necessarily involves placing two magnetometers in relative proximity which creates the potential for mutual interference.

TRACERS has two fluxgate magnetometers, the MAG science instrument (Strangeway et al. 2025) and the MAGIC technology demonstration (Miles et al. 2025a) mounted inboard. Both instruments are second-harmonic fluxgate magnetometers (Primdahl 1979). The two sensors mount on a common bracket and necessarily have some mutual magnetic interference due to their proximity. MAGIC, as a NASA NPR 7120.8 Technology Demonstration, is mandated to not interfere with the TRACERS host mission or degrade its science measurements. A combination of synchronizing the two instruments in hardware and careful characterisation of the stray magnetic field generated by each MAGIC sensor ensured that MAGIC’s impact on MAG could be removed via post processing.

### Sources of Fluxgate Magnetic Interference

**Fig. 8:**
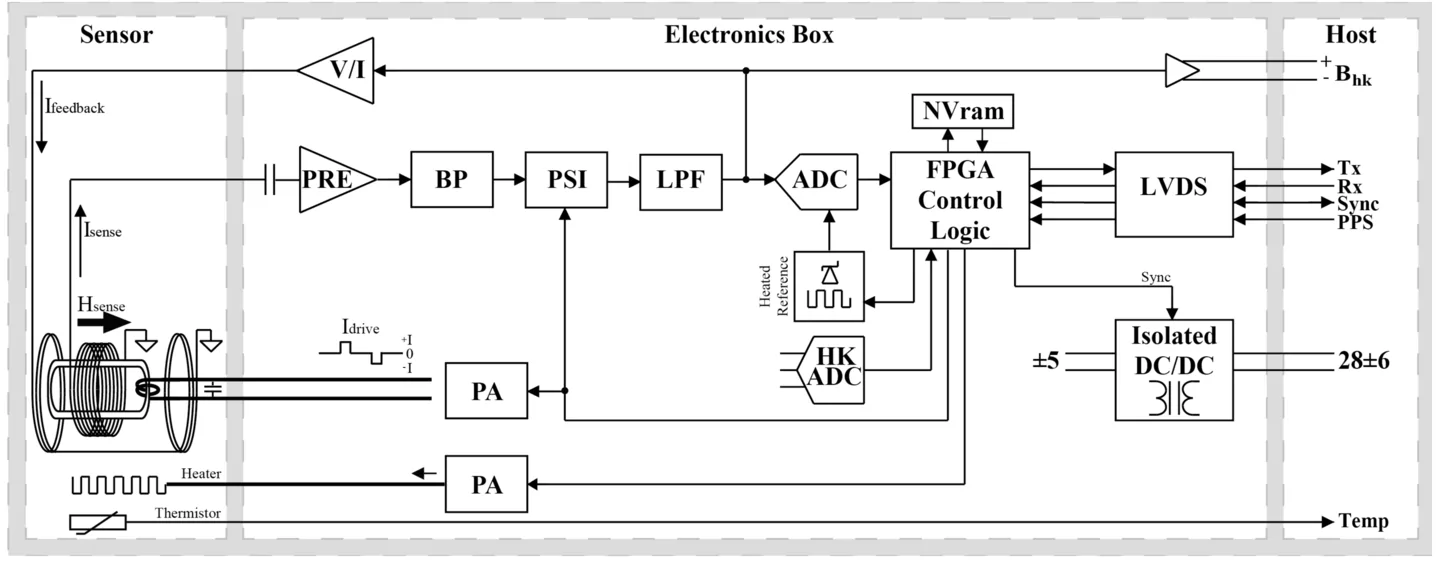
A single axis analog fluxgate magnetometer in the MAGIC instrument. The current $I_{\mathrm{drive}}$ periodically saturates a ferromagnetic core at frequency 1F creating a current $I_{\mathrm{sense}}$ at the second harmonic 2F that is proportional to the magnetic field. Quasi-DC current $I_{\mathrm{feedback}}$ is increased until it creates sufficient magnetic feedback to drive the local magnetic field in the solenoidal sense winding. From Miles et al. (2025a)

Fluxgate magnetometers (Primdahl 1979) sense the local DC and low frequency magnetic field by modulating (gating) it to AC and then measuring the voltage or current induced in a coil of wire called a sense winding. Figure 8 shows a single axis from the MAGIC fluxgate magnetometer. The current $I_{\mathrm{drive}}$ periodically drives a ferromagnetic core into magnetic saturation at 1F and in doing so generates an AC stray magnetic field at 1F. This modulates the effective permeability of the core and induces a current $I_{\mathrm{sense}}$ at the second harmonic 2F that is proportional to the local magnetic field. A quasi-DC current $I_{\mathrm{drive}}$ is increased until it creates sufficient magnetic feedback to drive the local magnetic field in the solenoidal sense winding to near-zero. This global negative magnetic feedback helps linearize the instruments response and extends the magnetic range but also generates a DC stray field proportional to the local magnetic field.

The operation of the fluxgate sensor creates magnetic interference relevant to a gradiometer application that can be grouped into four forms.

Each fluxgate core will concentrate the local magnetic field when its ferromagnetic material is unsaturated. Periodically, as the material is forced into magnetic saturation, the local magnetic field will relax as the core becomes functionally non-ferromagnetic. This periodic (2F) variation is partially mitigated by the magnetic field inside the sensor being held near-zero by the magnetic feedback of the instrument.

Stray field at (2F) is created directly by the drive winding used to force the core into magnetic saturation. The use of toroidal and quasi-toroidal windings helps mitigate this effect; however, the field outside the winding is non-zero particularly in the near field. This AC field is roughly constant, irrespective of the local magnetic environment, since it is dominated by the field created by driving the core into saturation. In practice, the two stray fields at 2F are difficult to separate and are both mitigated the same way.

Fluxgates typically use a bipolar drive current ($I_{\mathrm{drive}}$) to saturate the core to prevent the development of a remnant magnetic field that would affect the zero-offsets of the sensor. However, when the fluxgate is shut down the core is left having experienced some fraction of one phase of the drive magnetisation and can have a corresponding non-zero remnant magnetic field. This DC field varies in strength and polarity depending on where in the periodic drive sequence the instrument was shut down. In practice, this is the smallest of the interference mechanisms, and if the interfering instrument were permanently shut down would be removed by in-situ calibration.

Feedback current $I_{\mathrm{feedback}}$ creates magnetic feedback that nulls the field inside the sensor to near-zero. However, the feedback windings necessary also create a dipole-like stray field outside the sensor that varies proportional to $I_{\mathrm{feedback}}$. The resulting stray fields are quasi-DC and scale in proportion the local magnetic field because the magnetic feedback is trying to cancel the local field. In practice, this is the largest interference source. However, its behaviour is extremely predictable and can be removed with careful calibration via modification to the receiving instrument magnetic gain coefficient.

### Sensor Variants and Implications for Stray Fields

**Fig. 9:**
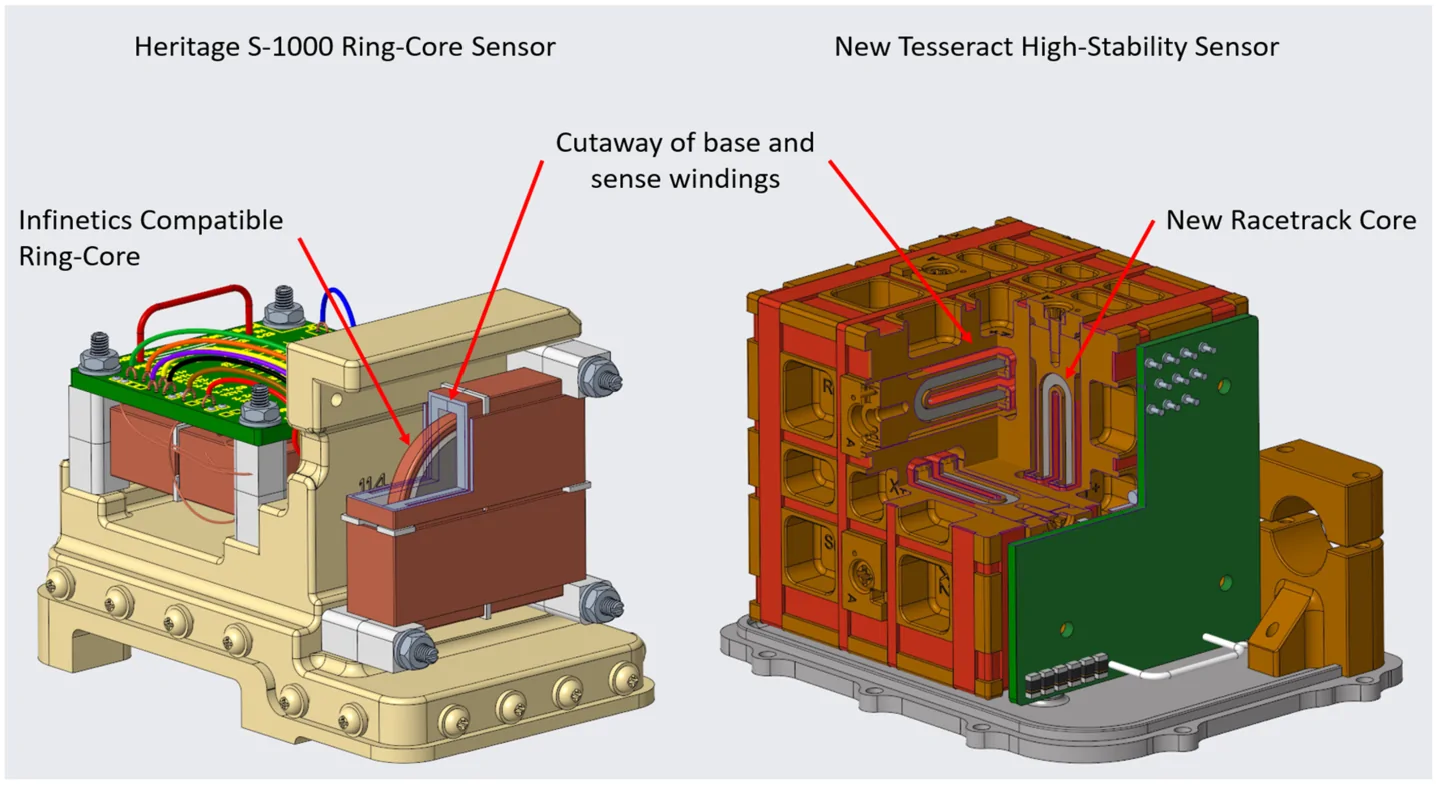
(Left) Exploded CAD model of MAGIC ring-core geometry sensor comprising two 2.54 mm ring-cores, each double wound with flat solenoidal sense/feedback windings. (Right) Exploded CAD model of the Tesseract geometry sensor comprising six racetrack cores, each with long-solenoid sense windings, and three external Merritt coil feedback windings. From Miles et al. (2025a)

The MAGIC technology demonstration uses a different sensor design on each of the two TRACERS spacecraft. The heritage ring-core sensor (Fig. 9a) uses 1” diameter cores compatible with the historical Infinetics S1000 design (Miles et al. 2019) and the new Tesseract (Greene et al. 2022) sensor (Fig. 9b) which utilizes new racetrack geometry fluxgate cores (Miles et al. 2022). The number, distribution, and geometry of the fluxgate cores and their respective drive windings influence the inter-sensor interference at the drive frequency. The difference in geometry and number of turns of the windings used to create magnetic feedback affect the geometry and intensity of the inter-sensor interference at DC.

#### The TRACERS Dual Fluxgate Gradiometer

**Fig. 10:**
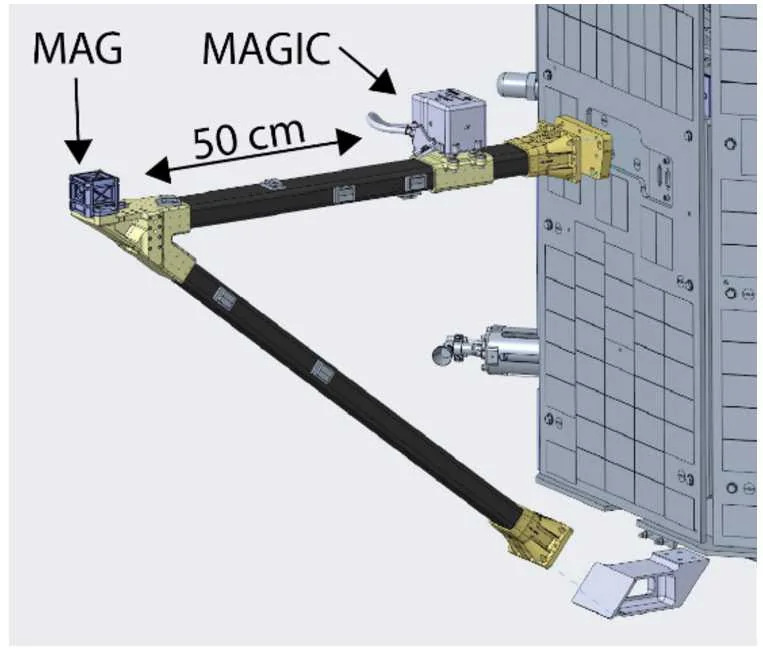
The primary MAG fluxgate sensor and the technology demonstration MAGIC sensor were mounted on a common boom with 50 cm separation

The baseline MAG science magnetometer was intended to be mounted at the end of a one meter long, two-segment magnetometer boom to distance it from the magnetic noise of the spacecraft with the MAGIC technology demonstration sensor mounted 50 cm inboard. However, due to a failure of the flight boom during environmental testing, both sensors were accommodated on a 70 cm fixed rigid bracket that preserved the 50 cm separation between the sensors (Fig. 10). The two instruments were designed to be oriented in the same coordinate system as the spacecraft via individual rotated brackets on the deployable boom that compensated for the deployment angle of the boom. However, this rotation was omitted in the shorter replacement bracket so that the two sensors were mounted both parallel and co-linear to provide additional symmetry to aid the gradiometer stray field removal in post processing.

### Quasi-DC Stray Field from Fluxgate Magnetic Feedback

The most significant interaction between the two fluxgates results from the global negative magnetic feedback action used by both magnetometers to extend their range and linearize their sensitivity. This effect was first simulated and then characterised in the lab to ensure that it could be corrected in post-processing. This work was completed before the mission switched from deployable booms to fixed brackets and therefore reflects the original sensor mounting orientations. However, the fundamental conclusion that the net effect is linear and manifests as a small change in the instrument sensitivity (∼5nT at 20,000 nT) remains valid and will be mitigated by on-orbit calibration.

Conveniently, the Bio-Savart model from Greene et al. (2022), developed to optimise the feedback field inside the Tesseract sensor, could be extended to evaluate the stray field generated outside the sensor. The model was later used to simulate the stray-field from the ring-core sensor as well.

**Fig. 11:**
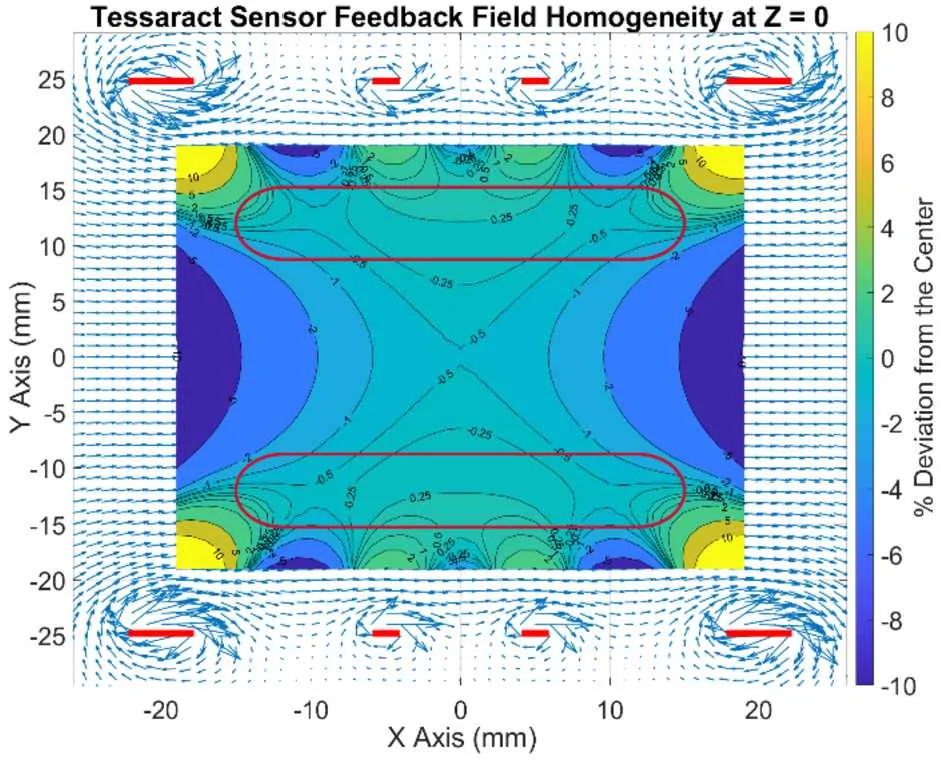
A two-dimensional cross section of the Boit-Savart model of the Tesseract sensor used to optimize the feedback field within the sensor Blue arrows map out the direction and magnitude of the B field, red rectangles represent the positions of the wires, and the dark red ovals show the location of the ferromagnetic fluxgate cores. From Greene et al. (2022)

Figure 11 shows a simulation of the magnetic field generated by the ‘Tesseract’ sensor’s feedback windings as a 2D slice. The Tesseract sensors feedback windings are arranged as a four-square Merritt winding, while Ringcore sensor uses a more traditional, continuous, toroidal feedback winding. The stray field from the Tesseract sensor was expected to be larger than that of the ring-core sensor due to its significantly larger Merritt coil feedback winding. In this simulation, each loop of wire in the feedback winding is modelled as a square array and assigned a length, a current, and a location on a 3D grid. A Biot-Savart integration is applied over each element in the square array to evaluate the magnetic field at every point on this grid. This process was repeated for every loop in the coil system and the resulting field values summed. The simulation was validated with magnetic measurements taken inside the prototype sensor with a DC gaussmeter probe. The boundaries of the simulation were then extended to estimate the MAGIC sensors’ feedback stray field experienced by the MAG sensor aboard each TRACERS spacecraft.

**Fig. 12:**
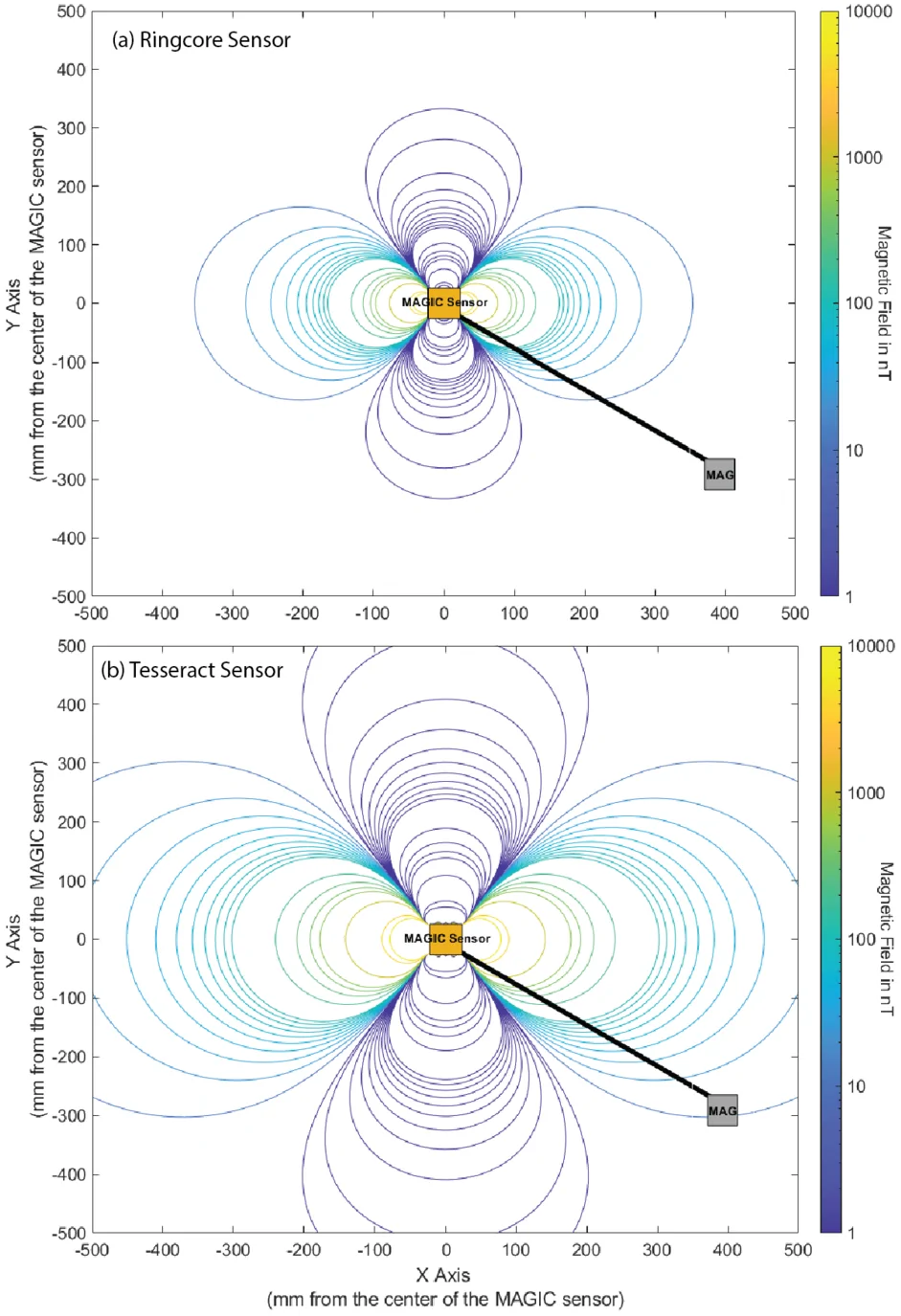
Biot-Savart model of the stray field generated by nulling 50,000 nT of field inside (a) the Tesseract sensor and (b) the ring-core sensor

The Biot-Savart model of the MAGIC sensors’ feedback windings was used to establish the worst-case upper limit on the strength of the MAGIC feedback stray field. The stray field from the MAGIC sensor feedback is proportional to the in-situ environmental field that is currently being canceled. Figure 12 simulates the feedback windings carrying a DC current that will null a 65,000 nT field inside the sensor. The stray field generated by each sensor is closely approximated by one dipole per sensor axis.

Magnetic feedback attempts to null the field inside the sensor, so the stray field generated by the sensor is linearly proportional to the applied external field. Table 3 shows the worst-case field generated by the magnetic feedback in each MAGIC sensor variant, responding a worst case where a 65,000 nT environmental field is aligned with a single sensor axis, as experienced by the MAG sensor at the end of the boom.

**Table 3:** Worst-case simulated stray magnetic field experienced by the MAG sensor at the end of the boom corresponding to 65,000 nT of environmental field aligning with a single axis of each sensor variant

Stray field	Ring-core Sensor +65,000 nT applied in each component			Tesseract Sensor +65,000 nT applied in each component		
	+X	+Y	+Z	+X	+Y	+Z
+X	−1.89	2.94	0	−10.67	16.87	0
+Y	2.95	−0.82	0	16.81	−5.22	0
+Z	0	0	4.01	0	0	11.74

MAGICs feedback field scales linearly with the Earth field so a ‘compensating gain coefficient’ $\boldsymbol{k}_{\boldsymbol{i}}$ is defined for each sensor axis i such that $B_{i,\mathrm{Actual}\ =} \mathbf{k}_{\boldsymbol{i}} * B_{i,\mathrm{Measured}}$. Where for the case of this simulation $B_{i,\mathrm{Actual}} = 65$,000 nT to simulate the most extreme case. Multiplying this coefficient by the measured field values from MAG compensates for the effects of the MAGIC sensor’s stray field and returns the undisturbed local field. Table 4 shows the compensating gain coefficients for the field measured by MAG. In practice, this is integrated into the instruments gain coefficient making the sensitivity determined for each sensor individually slightly different than that when the two sensors are mounted in flight configuration.

**Table 4:** Compensating gain coefficient $\boldsymbol{k}_{\boldsymbol{i}}$ required to correct each axis of the MAG sensor for the stray field resulting from magnetic feedback in the MAGIC sensor

Gain	Resulting from Tesseract			Resulting From Ring-core		
	X	Y	Z	X	Y	Z
$\mathbf{k}_{\boldsymbol{x}}$	1.000164154	0.999740462	1.000000000	1.000029077	0.999954769	1.000000000
$\mathbf{k}_{\boldsymbol{y}}$	0.999741385	1.000080308	1.000000000	0.999954615	1.000012615	1.000000000
$\mathbf{k}_{\boldsymbol{z}}$	1.000000000	1.000000000	0.999819385	1.000000000	1.000000000	0.999938308

### Experimental Validation of Stray Field from Magnetic Feedback

**Fig. 13:**
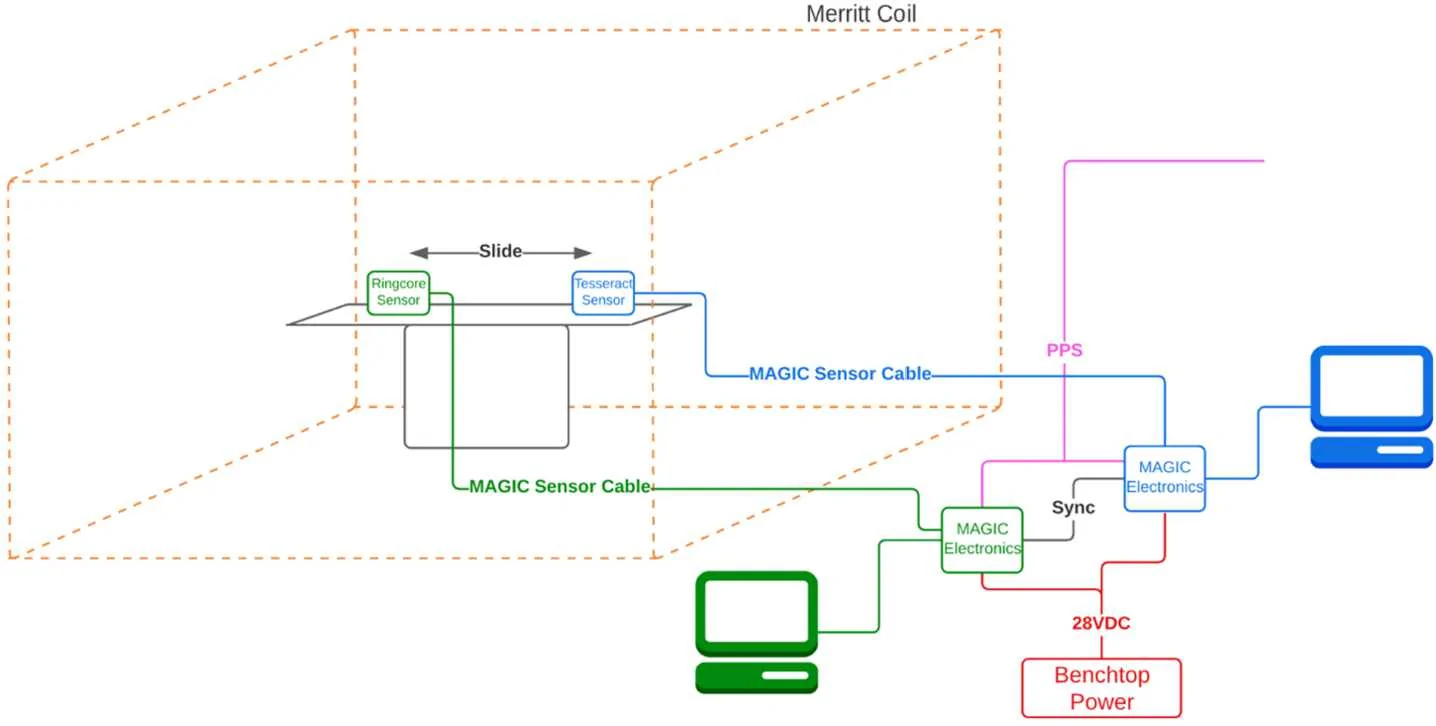
Schematic of experimental setup used to characterize the effect of MAGIC’s magnetic feedback on the measurements by the MAG sensor. External field was controlled using a two-meter three-axis Merritt coil. A sliding mechanical fixture was used to preserve the alignment between the two sensors but vary the separation to exaggerate the interference and provide robust characterisation

The model of the stray field resulting from magnetic feedback was validated using the test setup illustrated in Fig. 13. The MAG and MAGIC sensors were placed in a 2-meter Merritt coil with active magnetic compensation. Within the centre one-meter region of the coil system both sensors experience a common stimulus field to within one percent. The difference between the magnetic field measured by MAG with and without MAGIC applying magnetic feedback was measured at different applied field strengths and separations between the two sensors. This was tested with both the MAGIC Tesseract sensor and the MAGIC Ringcore sensor against the MAG sensor at separations of 8.5, 27.5, and 55 cm (measured from center to center). The difference was calculated from the field measured by MAG while toggling MAGIC’s magnetic feedback on and off in the direction aligned with the test field imposed by the Merritt coil.

**Fig. 14:**
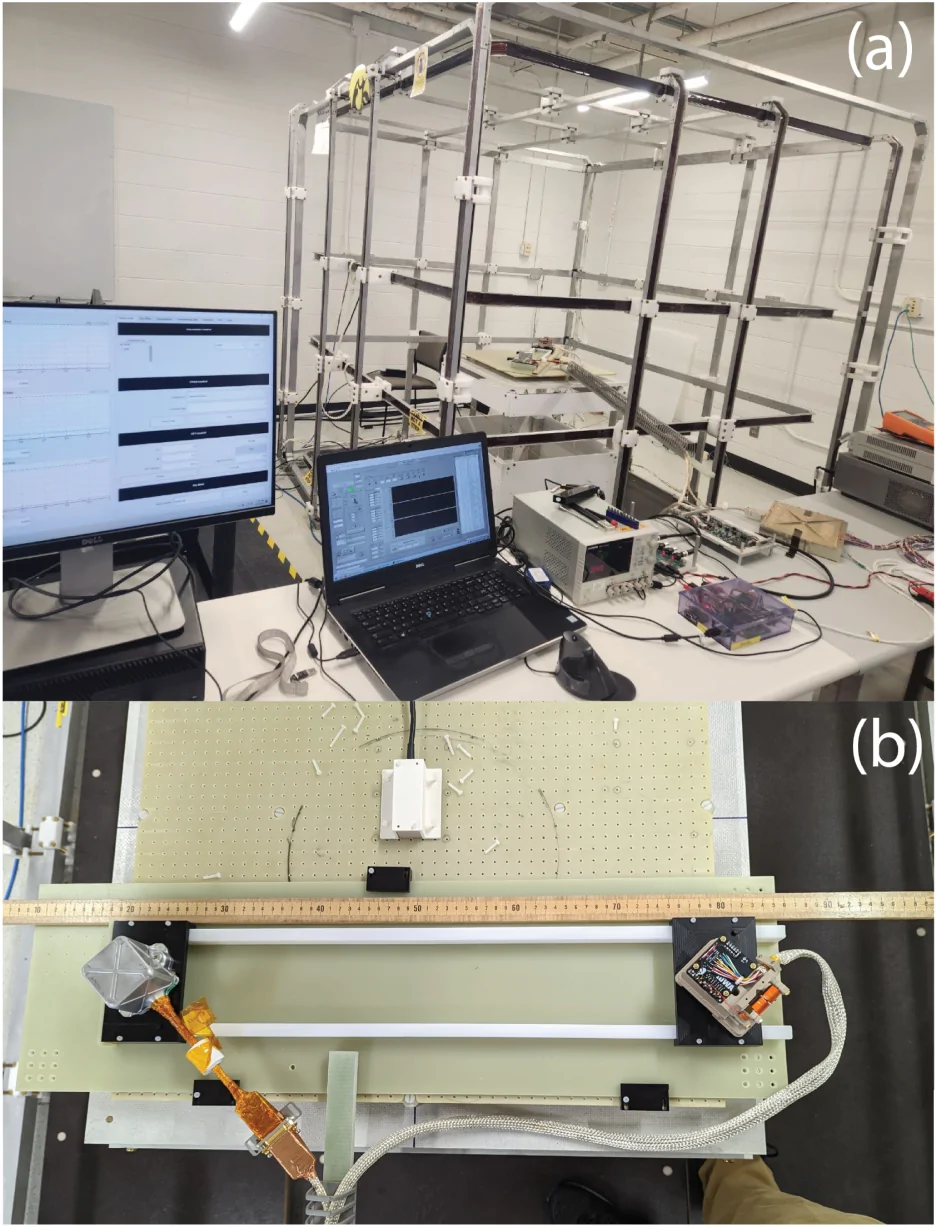
Picture of experimental setup using a two-meter Merritt coil test facility at the University of Iowa

Figure 14a shows the experimental setup where the two sensors were placed in the homogeneous test volume of the 2-meter Merritt coil facility at the University of Iowa. The electronics for both instruments and various test equipment were located nearby on ESD safe benching. Figure 14b shows the sensor fixturing for the experiment allowing the two sensors to be translated to various separations while preserving their relative orientation (this experiment was completed prior to the environmental test failure that necessitated moving to the fixed rigid bracket with a different sensor orientation).

**Fig. 15:**
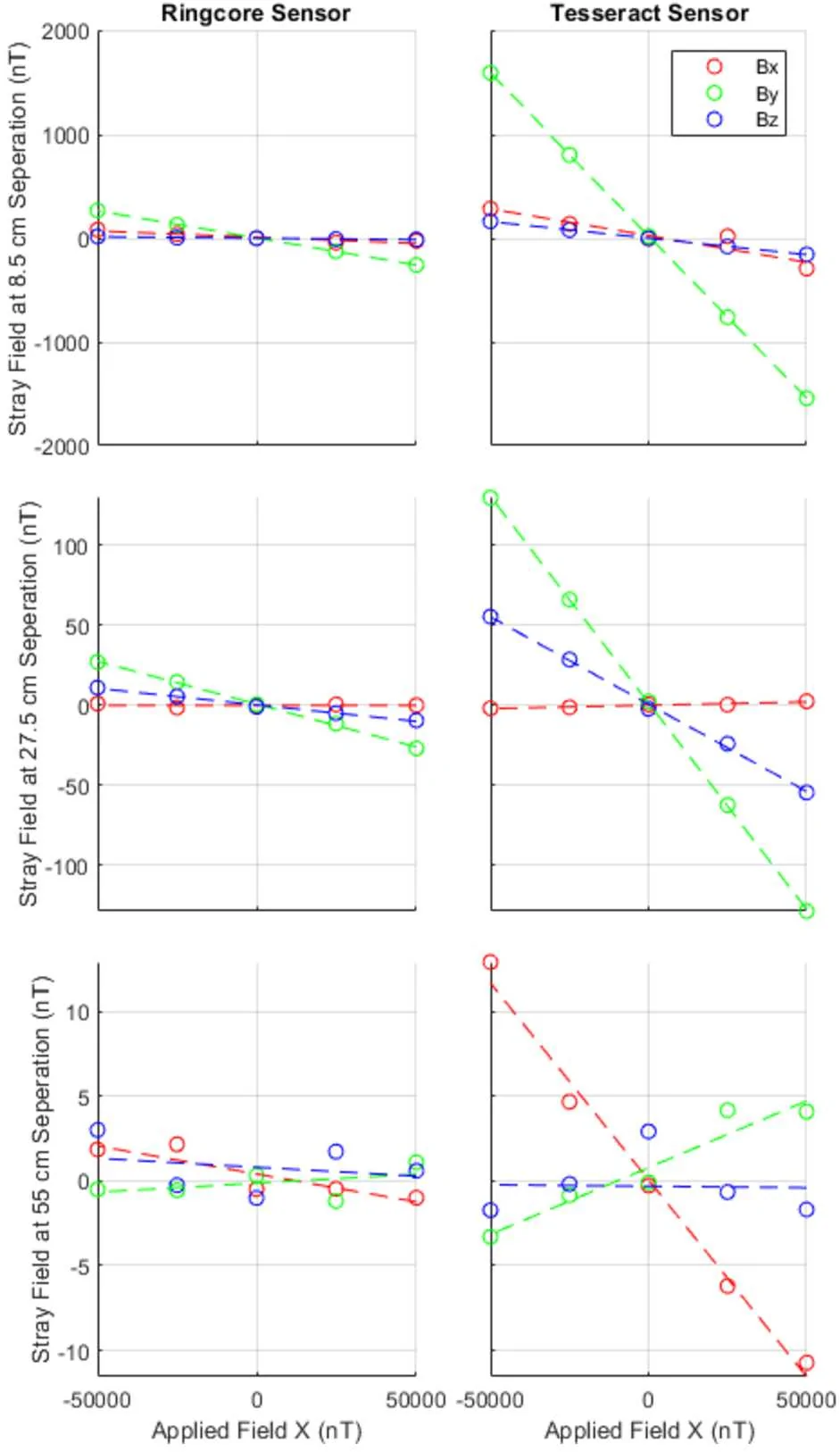
Measured stray field generated by magnetic feedback in the Tesseract and ring-core sensors. Note that while the intensity and polarity vary with the separation of the sensors, the effect is linear creating a predictable gain error in the second sensor

Figure 15 shows the results of this experiment at three different sensor separations of 55 cm (flight-like), 27.5 cm, and 8.4 cm (near field). In all conditions, the presence of a nearby fluxgate sensor with magnetic feedback enabled creates a linear gain error such that the degree of perturbation scales with the applied field. As predicted by the model, the Tesseract sensor with the larger area of feedback windings creates a measurably larger stray magnetic field.

The measured gain coefficients and the cross-axis coupling were all invalidated by the change from the deployable boom to the rigid brackets and, as the instruments were delivered before the bracket design was finalized, the testing could not be repeated. However, the robustly linear relationship will remain valid allowing the effect to be fully captured and mitigated via on-orbit calibration.

### Mitigating Fluxgate-Fluxgate Drive Signal Interference

**Fig. 16:**
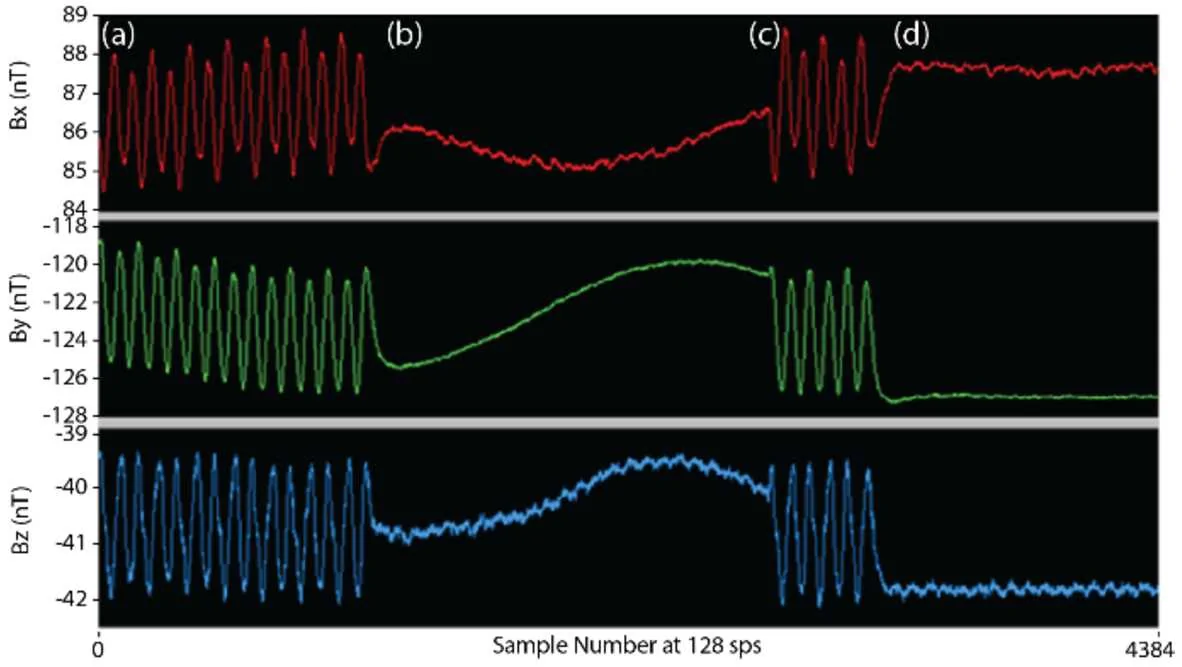
Beat frequency when the two sensors are (a) free running, (b) at close but non-synchronized frequency, (c) free running, and (d) synchronized

Each fluxgate must periodically drive its cores through magnetic saturation to modulate (gate) the local magnetic field and make a measurement. This creates a time-varying stray field whose manifestation depends on the relative phase of the core drive of the transmitting sensor and the sampling time of the receiving sensor. MAG and MAGIC both use a nominal 8192 Hz drive frequency so, due to their similar but not identical clock frequencies, this manifests as a beat frequency at the difference between the two clocks. Figure 16 shows data from an initial proof-of-concept test to mitigate this form of interference by synchronizing the instruments. Region (a) shows the two clocks free-running resulting in a rapid beat frequency. Region (b) shows the transmitting instrument set to a nearly identical, but not synchronized, frequency producing a much longer period beat. Region (c) shows another free-running interval followed by region (d) where the clocks are synchronized together. Further testing confirmed that, once synchronized, adjusting the relative phase of the two instruments adjusts the residual magnetic offset allowing a phase to be selected that minimizes the effect.

**Fig. 17:**
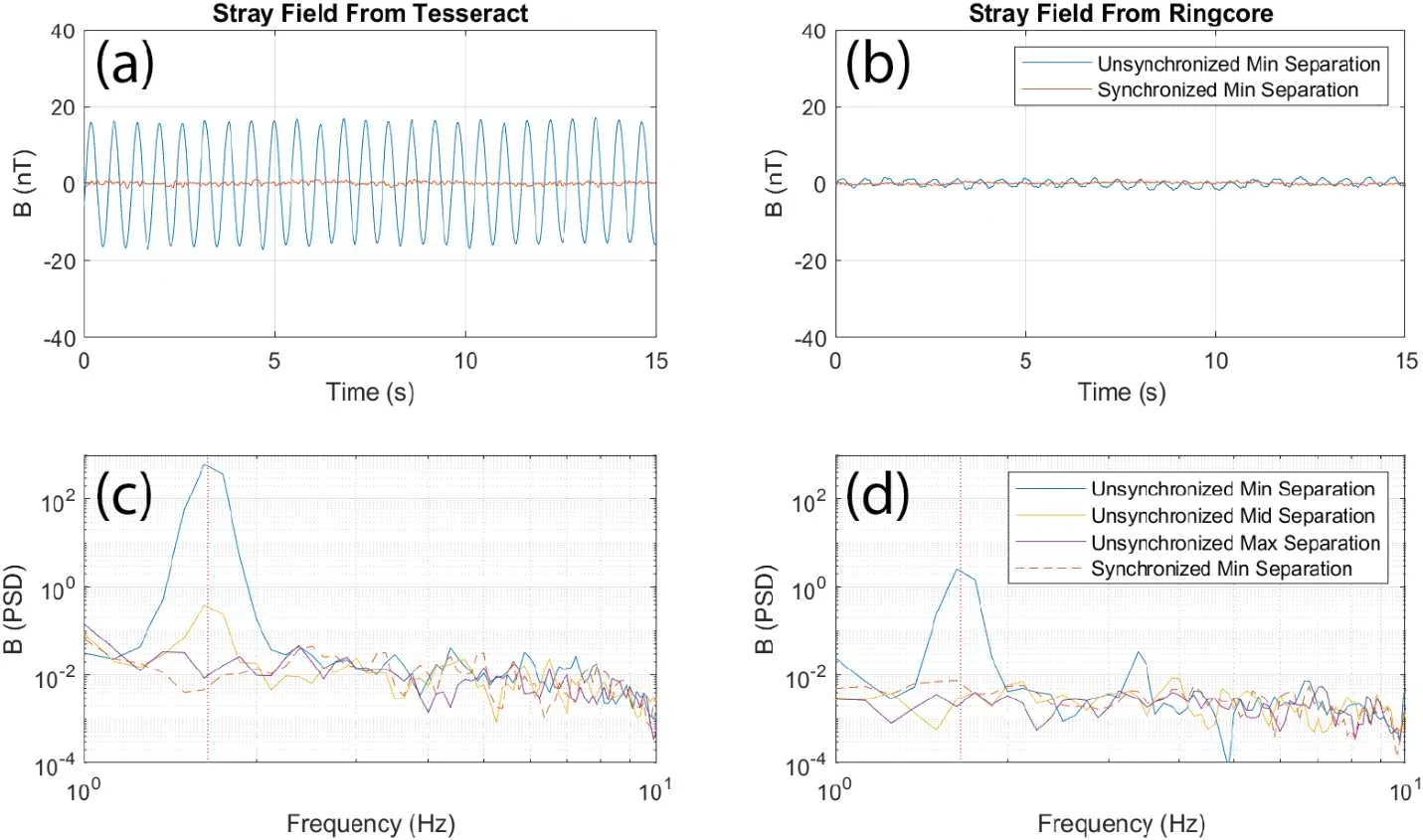
(a) Stray field from the Tesseract sensor at minimum separation with instruments running with and without synchronisation. (b) Same but showing stray field from the Ringcore sensor. (c) Unsynchronised stray field from the Tesseract sensor decaying with sensor separation. (d) Same but showing stray field from the Ringcore sensor

MAGIC implements a voltage-controlled crystal oscillator as its base clock and uses a Pulse-Width-Modulated (PWM) signal driven by Proportional-Integral-Differential (PID) control loop to steer the oscillator until its local 1F signal matches a 1F sync signal from MAG in both frequency and phase. Figure 17 shows the result for the stray field generated by the Tesseract sensor (a, c) and the ring-core sensor (b,d). This data is taken at minimum separation (the two sensors directly adjacent) to enhance the effect and check the effectiveness of the mitigation. The Tesseract uses race-track cores with quasi-toroidal drive windings (Miles et al. 2025a), which likely explains its significantly larger stray field compared the closed toroidal winding of the S1000 compatible ring-core. Regardless, synchronisation fully mitigates this effect for both sensor variants as shown in both the time series (a,b) and in spectral analysis (c,d).

## Swing Test to Validate DC Stray Field

A swing test was used to estimate the static magnetic field of each TRACERS spacecraft in its unpowered configuration. This procedure was based on a similar one used to measure the static magnetic field for the Van Allen Probes mission (Kirby et al. 2013). Each spacecraft was evaluated in two ways. The first involved a shallow swing of the suspended spacecraft with the resulting perturbation of the local magnetic field measured by static magnetometers on tripods at fixed distance. The second test measured the angular dependence of the stray spacecraft field by rotating the spacecraft next to stationary magnetometers.

**Fig. 18:**
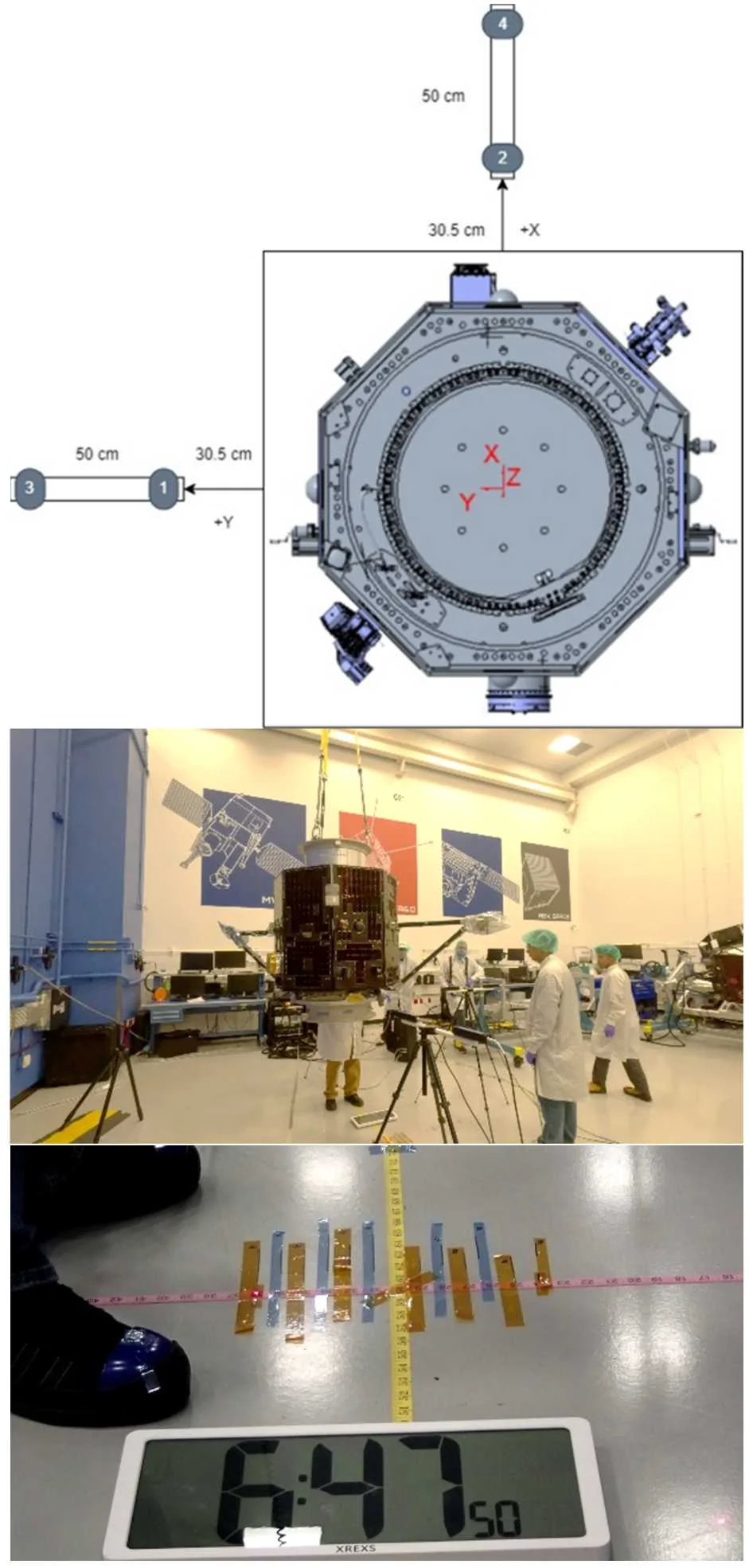
Experimental setup for the TRACERS swing tests

The spacecraft was suspended from a crane with the hook as far above the spacecraft as possible within the integration high bay. Figure 18 shows the test configuration with two magnetometers, numbered as 1 and 3 at 30.5 and 80.5 cm separation from +Y side of the spacecraft. Magnetometers 2 and 4 were at the same distance from the +X side of the spacecraft.

Measurements were acquired continuously as the spacecraft slowly swung with a maximum displacement of about 15 cm. The spacecraft was hung over a reference point, aligned by a downward pointing laser pointer, and the degree of swing was captured by video as the laser spot traversed a set of marked distances. Synchronized measurements from the four magnetometers captured the time varying local magnetic field as it was modulated by the moving stray field of the spacecraft. A simple dipole model was fit to this data to estimate the stray DC field of each spacecraft.

**Fig. 19:**
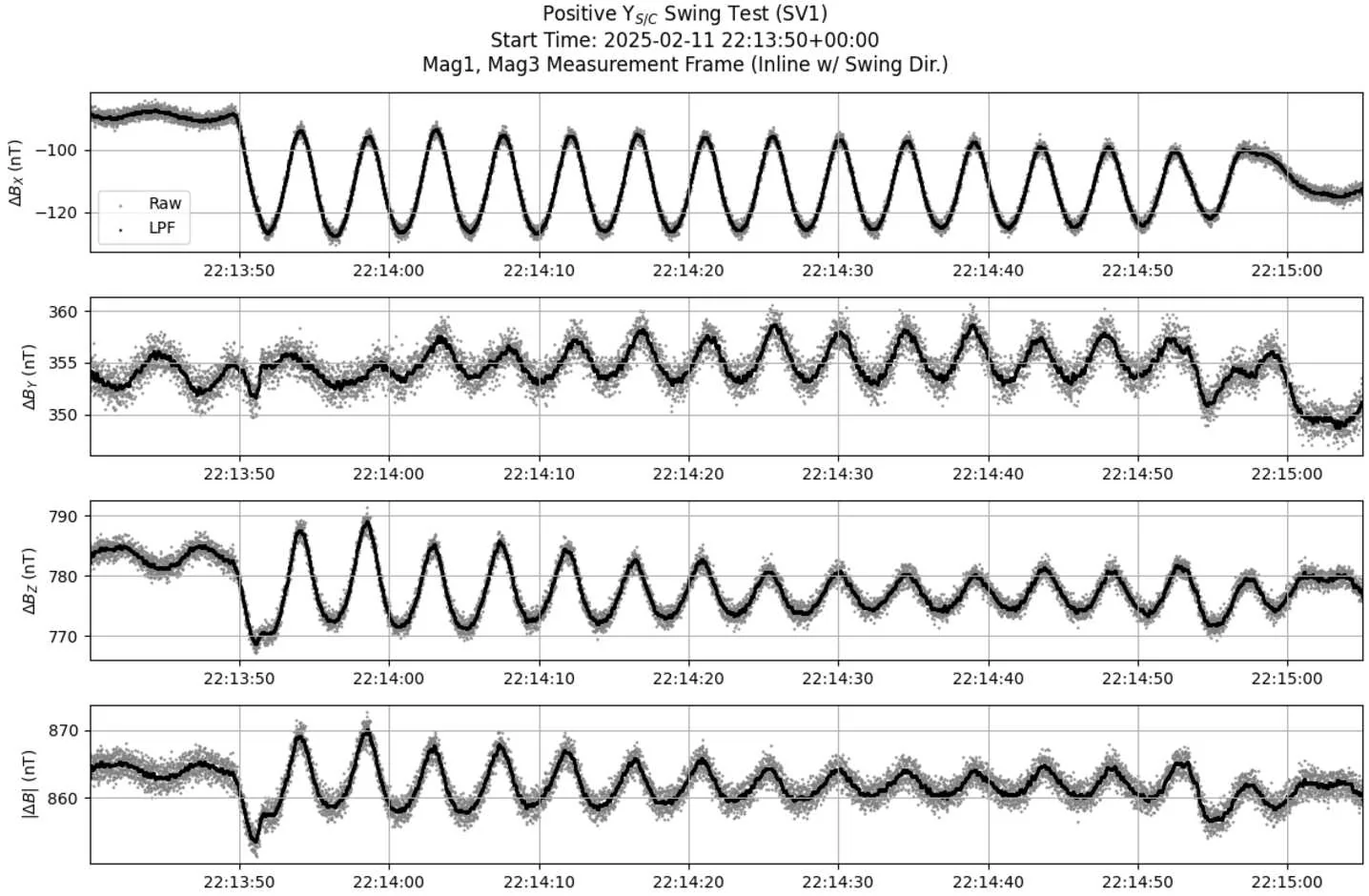
Magnetic field gradient measured in-line with the swinging spacecraft

Figure 19 shows the magnetic field gradient, measured at 125 Hz, between the pair of magnetometers located along the Y axis of the spacecraft body during the magnetic swing test. The top three panels are the respective X, Y, and Z axis gradients between the inline magnetometer pair, and the bottom panel shows the magnitude of the axial gradients. Points in gray are the raw values, whereas points in black are the result of application of a fifth-order 5 Hz low pass filter. The start of the damped sinusoidal oscillation shortly before 22:13:50 marks the beginning of the spacecraft’s swing and corresponds to the measurement made closest to this gradiometer pair. As the test operator handling the spacecraft releases the vehicle and steps away from the magnetometers the data becomes smoother and more sinusoidal (i.e., by 22:14:05). At approximately 22:14:55 the test operator moves closer to the magnetometers to resume control of the spacecraft, completing the test. This test was repeated several times to ensure repeatability of analysis.

Controlled spacecraft rotation in the same experimental setup provided the azimuthal variation in the spacecraft’s stray magnetic field. The suspended spacecraft was rotated by hand and was turned one way, and then immediately referenced for validation and error estimation. The environmental field was checked immediately before and after each test to confirm that no large environmental changes that would contaminate the measurements.

The reduction and synthesis of this data into stray field estimates at the sensor location was challenging due to variations in environmental magnetic field and strong gradients in the local field, resulting in preliminary uncertainties on the order of 50%. To overcome this, a new analysis has been developed that fits the gradients measured by the two pairs of sensors. The results of this work, forthcoming in a separate publication, significantly improve this uncertainty and suggest that the control threshold of 100 nT at the sensor was met with margin.

## Adapting to Late Accommodation Changes

**Fig. 20:**
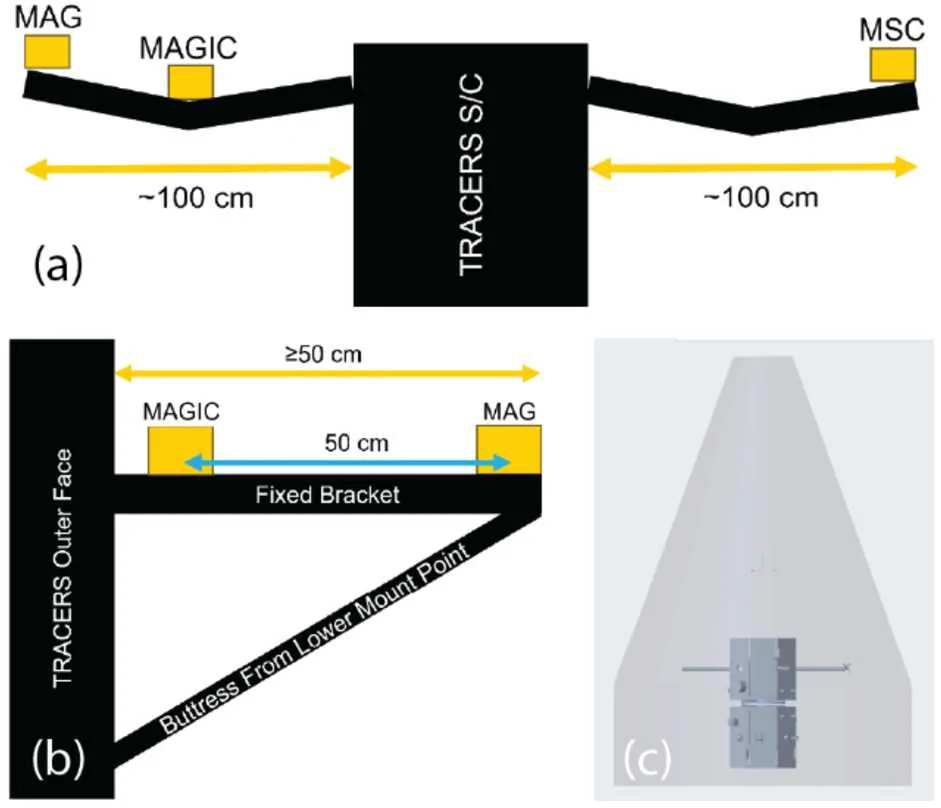
(a) TRACERS baselines design with the MAG fluxgate sensor and the MSC search coil sensor each mounted on opposing 100 cm deployable booms. The MAGIC technology demonstration fluxgate sensor was mounted on the outboard segment of the MAG boom near the elbow joint. (b) Contingency design using a fixed rigid bracket with the MAG sensor at the tip and the MAGIC sensor 50 cm away from the MAG sensor. (c) The bracket contingency design was enabled by the volume allowed around the TRACERS spacecraft when accommodated as a “Cake-Topper” payload on a SpaceX Falcon 9

The TRACERS DC fluxgate magnetometer (MAG) sensor and the AC search coil magnetometer (MSC) sensors were designed to be accommodated at the end of opposing 100 cm deployable booms as shown in Fig. 20a. However, a flight boom failed during vibration testing leading TRACERS to adopt fixed rigid brackets (Fig. 20b) that were enabled by the large usable volume within the Falcon 9 fairing as TRACERS had been selected to occupy the top “cake-topper” position (Fig. 20c). Due to schedule constraints, this solution was accepted when initial analysis showed that the MAG and MSC sensor could be accommodated at least 50 cm from the spacecraft surface with the intention of mitigating the apparent increase in magnetic noise, due to the reduced separation, using signal processing.

### Science Impact of Increased Magnetic Noise

TRACERS has three science questions (Miles et al. 2025b; Petrinec et al. 2025) that are not equally affected by increased magnetic noise:

SO1: Determine whether magnetopause reconnection is primarily spatially or temporally variable for a range of solar wind conditions.

SO2: For temporally varying reconnection, determine how the reconnection rate evolves.

SO3: Determine to what extent dynamic structures in the cusp are associated with temporal versus spatial reconnection.

SO1 and SO2 require the in-situ $\boldsymbol{E} \times \boldsymbol{B}$ convection velocities. However, because the background field is large (∼50,000 nT), the accuracy of the derived velocity (∼1%) is dominated by the uncertainty of the electric field even with significant stray magnetic fields. This was demonstrated on the TRICE-II suborbital rocket (Petrinec et al. 2023; Sawyer et al. 2021; Trattner et al. 2025) using a body-mounted engineering magnetometer. To this end, TRACERS is required to measure the three component DC vector magnetic field with an accuracy of 100 nT at a temporal resolution of 0.1 sec.

S03 requires the magnitude and direction of the large (∼50,000 nT) background field to orient pitch-angle distributions of the electron and ion measurements. This can also be done using a body-mounted magnetometer due to the comparatively coarse angular bins of the particle instruments. However, SO3 also requires the characterization of Field-Aligned-Currents (∼100 nT) and Alfven waves (∼1 nT) which are more sensitive to stray magnetic fields. To fully characterize the wave processes, TRACERS must measure the three component AC magnetic fields to up to 1000 Hz.

### Noise Mitigation

The stray magnetic field experienced by the TRACERS magnetometers are expected to comprise three classes as shown in Table 5. These types of magnetic interference are common on space physics missions and existing algorithms can remediate them to varying degrees.

**Table 5:** Expected classes of stray magnetic fields on TRACERS and expected mitigation by various post-processing techniques

Stray Field Type	Frequencies	Expected Post Processed Mitigation
Static	0 Hz	10-100× via calibration
Solar Panels	∼0.16 Hz	10× via spin fit and calibration
Dynamic Systems	∼1 to ∼1000 Hz	10× via statistical signal processing and machine learning

The static stray fields due to magnetized and ferromagnetic materials in the spacecraft are effectively a static offset that can be characterized both before launch, using the planned spacecraft swing test, and on-orbit using in-situ calibration. Recent works have successfully characterized and subtracted these static fields by factors of 10-100 times (e.g., Wallis et al. 2015; Broadfoot et al. 2022).

**Fig. 21:**
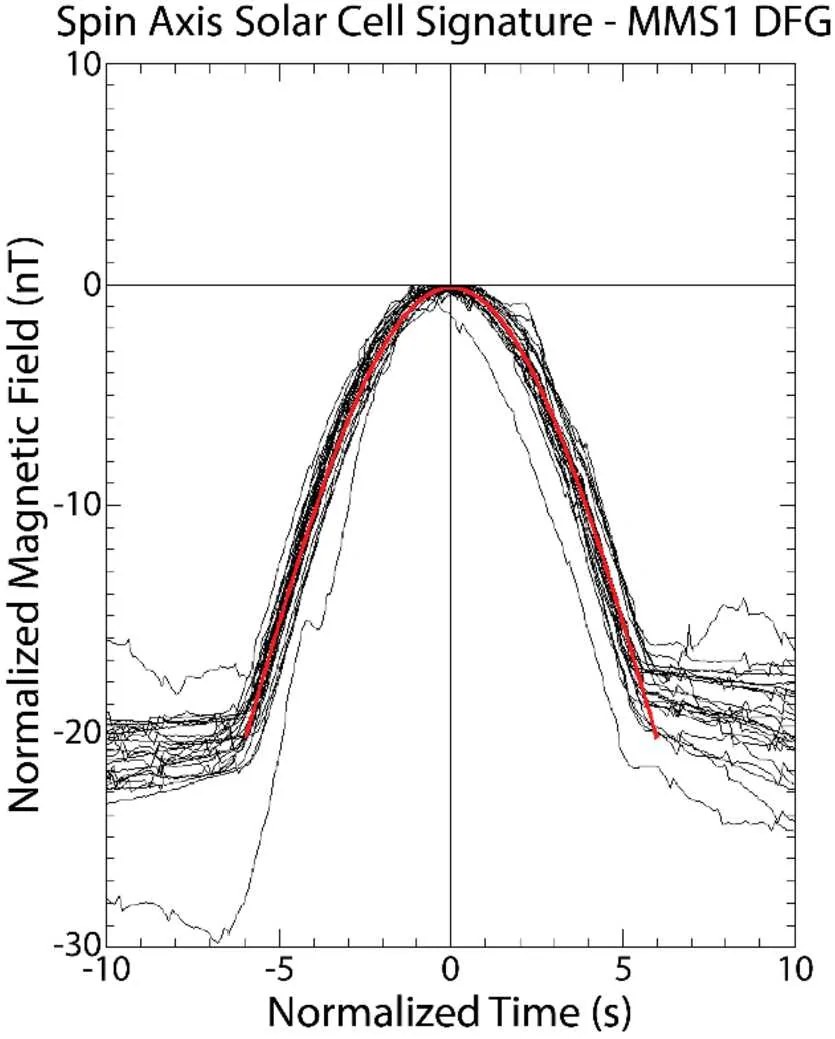
Superposed epoch analysis of the stray magnetic field component in the spin axis direction from the MMS solar panels prior to the magnetometer boom deployment (black) compared to a cosine fit (red)

Stray fields from the solar panels are more challenging to remove as they are modulated at the spacecraft spin frequency. However, since the stray field from the solar panel current follows the solar illumination angle as the spacecraft spins, it can be robustly fit to a half-waveform cosine and subtracted from the measured data. Figure 21 shows an example from MMS prior to the deployment of the magnetometer boom (analogous to bulkhead-mounting the sensor). An average cosine fit (red) matches the solar panel stray field (black) to better than 10 times.

Dynamics stray fields due to spacecraft subsystems or other instruments are generally the most challenging to remove in post-processing as they tend to vary in both amplitude and frequency and can overlap valid geophysical signals. However, the discipline of magnetic field signal processing and data cleaning has advanced significantly since TRACERS was conceived (Constantinescu et al. 2020; Finley et al. 2023a, 2023b; Hoffmann et al. 2024; Hoffmann and Moldwin 2023; Imajo et al. 2021; Ponder et al. 2016; Ream et al. 2021; Sen Gupta and Miles 2023; Sheinker and Moldwin 2016). Singular spectral analysis (SSA) techniques (Finley et al. 2023b, 2023a) are general purpose enough that, without optimization, algorithms developed for the e-POP/Swarm-Echo spacecraft (Wallis et al. 2015; Yau and James 2015) were effectively applied to Parker Solar Probe (Raouafi et al. 2023) and have mitigated dynamic noise sources by 89-95%. A co-investigator with significant signal processing experience was added to assist in the post-launch AC noise removal.

**Fig. 22:**
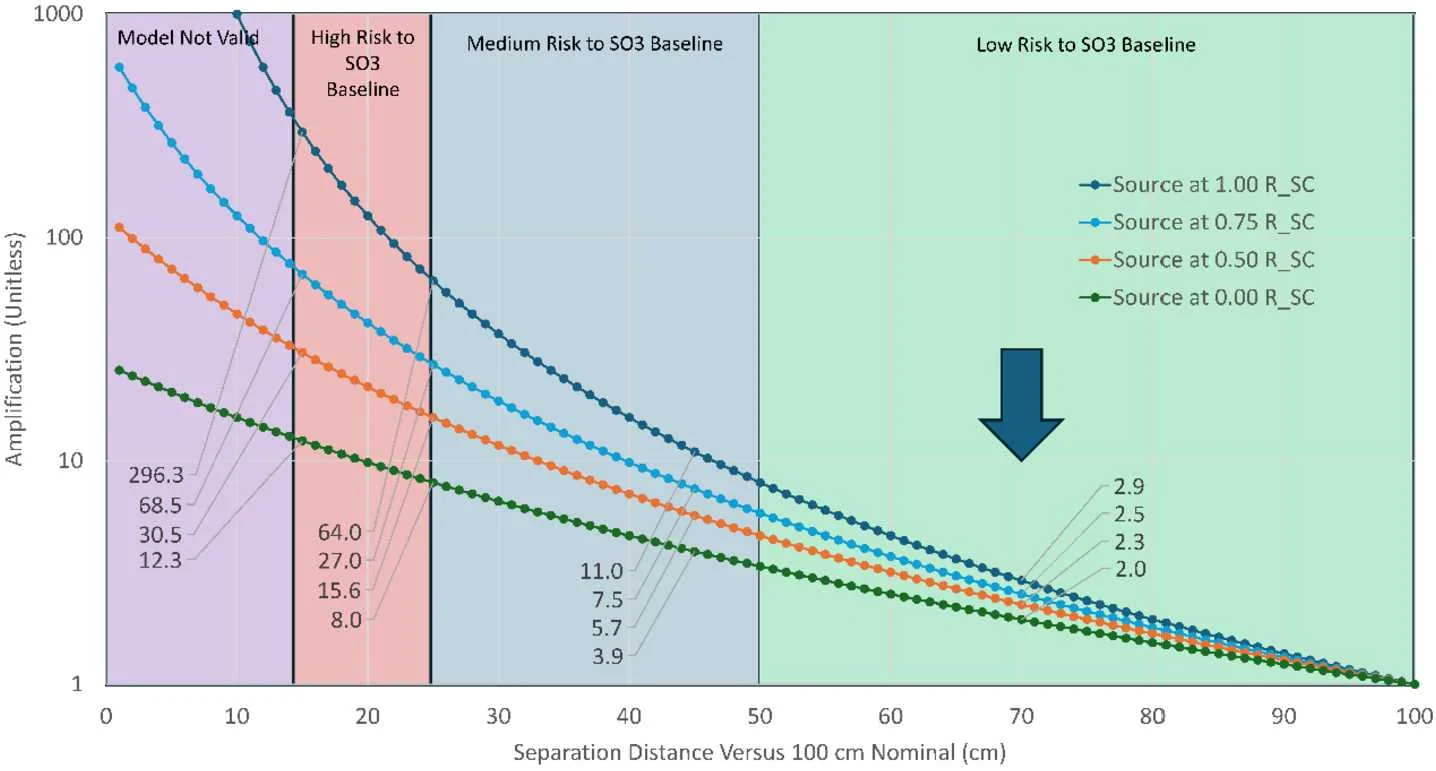
Effective magnetic noise amplification of moving the magnetometer sensors closer to the spacecraft compared to the baseline 100 cm location. The amplification is calculated using dipole projection with sources ranging from the center of the spacecraft $\left ( 0.00\ R_{SC} \right )$ to the outer face $\left ( 1.00\ R_{SC} \right )$. The dipole model will be invalid near the spacecraft due to non-negligible multipole terms

Although each noise class will need to be mitigated using a different post-processing technique, all three classes have been mitigated to less than 10% of their original value using existing algorithms. TRACERS can therefore tolerate an increase of the stray magnetic field at the sensors of up to ∼10 times by the application of post-processing using existing algorithms. Stray magnetic fields drop off as a dipole $\left ( {1} / {r^{3}} \right )$ or faster so moving the magnetometer sensors in towards the spacecraft will effectively amplify the stray fields. Figure 22 shows the approximate predicted apparent noise amplification as the sensors are moved inward, normalized to the noise with the sensor at the original location 100 cm from the spacecraft surface. The four lines bound the cases of noise source locations ranging from the center of the spacecraft $\left ( 0.00\ R_{SC} \right )$ to the spacecraft surface $\left ( 1.00\ R_{SC} \right )$. The apparent amplification of the spacecraft’s stray magnetic field is less than ten times (∼4-11 times) at lengths of ∼50 cm so that was adopted as the threshold that should be able to be mitigated by existing signal processing algorithms.

A bracket was designed that achieved a 70 cm distance to the tip-mounted sensors using the existing mounting interfaces and without requiring any redesign of the spacecraft or significantly altering the assembly, integration, and test flow. At this length, the effective noise amplification is modest (∼2-4×) and could likely be fully remediated with existing signal processing techniques. This design change was assessed to not impact SO1, SO2, or the Threshold closure of SO3. However, it likely carries a low likelihood but medium consequence risk to the baseline closure of S03. Specifically, if the algorithms to remove the time-varying stray fields significantly underperform what has been achieved in comparable applications then the magnetic component of the Alfven waves may not be sufficiently captured, resulting in the presence of waves having to be inferred, via assumptions, from the AC electric field alone.

## Conclusions and Future Work

The TRACERS mission used a combination of design best-practices, magnetic screening of subsystems, and characterisation of the as-built spacecraft to provide a magnetic environment that should be sufficient for the magnetic measurements required for its science objectives. The success of these processes will be evaluated once both spacecraft are on-orbit. The offsets recovered by in-situ magnetic calibration of the fluxgate magnetometers will validate the effectiveness of the DC magnetic cleanliness program and the magnetic swing test. Any significant AC magnetic sources will be compared to the catalog created during integration with the goal of identifying the source subsystem or activity and developing and algorithm to mitigate the noise by operational constraint or post-processing.

## Appendix: Magnetically Preferred Materials

Table 6 is based on heritage guidance derived from materials selection practices for magnetically clean spacecraft including the GSFC Materials Engineering Branch Technical Information Paper No. 128 (West et al. 1971) and related magnetic cleanliness studies. These sources established initial material classification driven primarily by known magnetic behavior of material systems, particularly the presence of ferromagnetic constituents (e.g., Fe, Ni), as well as known sensitivities to processing-induced magnetization. Materials are intended to be initially categorized qualitatively for design guidance, but final acceptability depends on their integrated contribution to the system magnetic environment.

**Table 6:** Materials exhibiting preferred and non-preferred magnetic properties

Exhibits non-magnetic properties (Preferred)	Exhibits Magnetic Properties (Non-Preferred)
* Alloy 30, 60, 90	Alloy 426
Aluminum3	Alloy 7201
Beryllium (not for primary structure)	Carbon Steel (all types)
AlBeMet (not for primary structure)	Chromium
Copper	Cobalt
Delrin	Copperweld
Electroless Nickel (10%-13% weight high-phosphorous)	Dumet
* Elgiloy	Electroless Nickel (except high-phosphorous)
Germanium	Electroloy
Gold	Elinvar
* Inconel 600, 625, 7184	Fenicoloy
Lead	Ferrites
Manganin	Gridaloy M, P
* Moleculoy	Haynes Alloy #6
Molybdenum	Invar
* Neutroloy	Iron
* Nickel Silver	Kovar
Nylon	Mesoloy
Silicon	Molypermalloy
Silver (avoid use when directly exposed to space, i.e. atomic oxygen)	Monel K1
* Stainless Steel 310	Monel R
* Stainless Steel 316	Mumetal
* Stainless Steel A286	Nichrome
Tantalum	Nickel 200, 270
* Nitronic 60	Nickel Iron
Titanium	Pelcoloy
Tungsten (alloys shall not contain iron)	Permalloy
Ultem	Platinum
Zinc	Remendur
Zirconium	Rodar
Polyether ether ketone (PEEK)	Stainless Steel 2022
Alumina (aluminum oxide)	Stainless Steel 3022
Macor	Stainless Steel 3032
Shapal	Stainless Steel 3042
Silicon Nitride	Stainless Steel 403 & 405
Aluminum Nitride	Stainless Steel 410 & 416
G10/FR4	Stainless Steel 430 & 446
Vespel	Stainless Steel 15-5PH and 17-4 PH
	Stainless Steel AISI 440C
	Supermalloy
	Ti 430
	Vicalloy
